# Pharmacological Potential of Betulin as a Multitarget Compound

**DOI:** 10.3390/biom13071105

**Published:** 2023-07-12

**Authors:** Feyisayo O. Adepoju, Kingsley C. Duru, Erguang Li, Elena G. Kovaleva, Mikhail V. Tsurkan

**Affiliations:** 1 Department of Technology for Organic Synthesis, Chemical Technology Institute, Ural Federal University, Mira 19, 620002 Yekaterinburg, Russia; besee010@gmail.com (F.O.A.); e.g.kovaleva@urfu.ru (E.G.K.); 2 Center for Advanced Biotechnology and Medicine, Rutgers University, Piscataway, NJ 08854-8021, USA; 3 Medical School, Nanjing University, Nanjing, 22 Hankou Road, Nanjing 210093, China; erguang@nju.edu.cn; 4 Leibniz Institute of Polymer Research, 01069 Dresden, Germany

**Keywords:** betulin, inflammation, anti-inflammatory, hepatoprotective, natural products (MAPK, Nrf2, NFκB)

## Abstract

Betulin is a natural triterpene, usually from birch bark, known for its potential wound-healing properties. Despite having a wide range of pharmacological targets, no studies have proposed betulin as a multitarget compound. Betulin has protective effects against cardiovascular and liver diseases, cancer, diabetes, oxidative stress, and inflammation. It reduces postprandial hyperglycemia by inhibiting α-amylase and α-glucosidase activity, combats tumor cells by inducing apoptosis and inhibiting metastatic proteins, and modulates chronic inflammation by blocking the expression of proinflammatory cytokines via modulation of the NFκB and MAPKs pathways. Given its potential to influence diverse biological networks with high target specificity, it can be hypothesized that betulin may eventually become a new lead for drug development because it can modify a variety of pharmacological targets. The summarized research revealed that the diverse beneficial effects of betulin in various diseases can be attributed, at least in part, to its multitarget anti-inflammatory activity. This review focuses on the natural sources, pharmacokinetics, pharmacological activity of betulin, and the multi-target effects of betulin on signaling pathways such as MAPK, NF-κB, and Nrf2, which are important regulators of the response to oxidative stress and inflammation in the body.

## 1. Introduction

Natural products have long been regarded as an attractive source of pharmacologically active substances, especially for infectious diseases and cancer. Despite the adoption of synthetic chemistry-based approaches in pharmaceutics, natural products continue to make significant contributions to the prevention and treatment of diseases. This is because of their diversity in nature, complexity, novelty, low toxicity, and broad efficacy. Beyond that, there is a need to uncover new compounds that can effectively treat diseases to improve therapeutic options [1]. Triterpenes are a class of natural compounds widely distributed in plants, with potential anti-inflammatory, hepatoprotective, antioxidant, anti-cancer, anti-viral, or cytotoxic properties. However, they usually account for less than 0.1% of plant organs’ dry weight.

**Figure 1:**
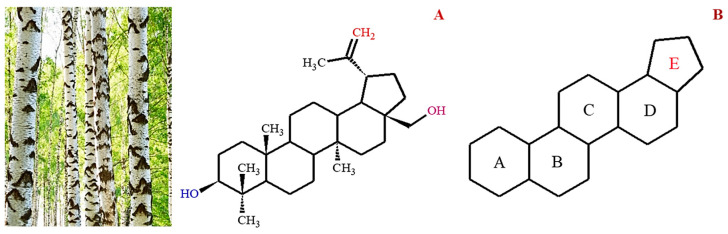
Image of birch trees which is the main source of betulin. The structural formula of betulin (**A**) and lupane pentacyclic skeleton of betulin (**B**).

Betulin, chemically known as lup-20(29)-ene-3β,28-diol, is a naturally occurring triterpene characterized by a five-membered ring and an isopropylidene group (Figure 1). Betulin and its other derivatives are found in various plant species, especially in the Betulaceae family, which is responsible for the silvery color of birch trees [2]. Factors such as species, geographical location, age, and climate have been shown to influence the dry weight content of betulin (10–45%) in plants. Considering the abundance of betulin in nature, other potentially bioactive forms are typically prepared using simple oxidation-reduction protocols, such as Jones oxidation and Pinnick oxidation.

**Figure 2:**
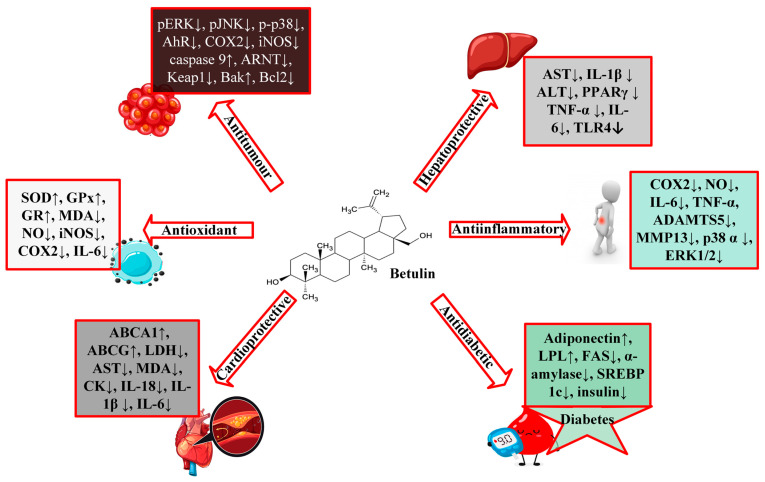
A schematic representation of the biological activities of betulin.

According to the available literature, it is evident that betulin has a broad spectrum of biological activities (Figure 2), including anti-HIV, anti-fungal, anti-bacterial, anti-inflammatory, anti-tumor, anti-leishmania, and immune regulatory effects [1,3,4]. However, most of these studies have focused on derivatives of betulin. Recent studies have shown that betulin exerts significant pharmacological effects once its insolubility is resolved. From these studies, betulin is shown to exert multitarget activities in different organs and disease states. Betulin inhibits proinflammatory cytokines (IL-6, IL-1β, TNFα), HMGB-1, NFκB, and MAPK, which results in the reduction of lung and liver injuries in septic rats [5]. Even though the exact molecular mechanism of these activities is still unknown, studies have purported that the anti-inflammatory properties of betulin are at the heart of its different biological functions [5]. Many diseases, including infection, cancer, allergies, diabetes, asthma, arthritis, and atherosclerosis, are characterized by chronic inflammation [6,7]. Since inflammation is a key process in the development and occurrence of many chronic diseases, compounds with multitarget properties are the direction of the search for therapeutic drugs.

Despite diverse biological and therapeutic activities, the molecular mechanisms of betulin as a multitarget anti-inflammatory compound remain unclear. This is because there are many possible pharmacological targets in the inhibition of inflammation, including chemokines, cytokines, and pro-inflammatory transcription factors. Hence, the current review aims to discuss the relevant studies that propose the therapeutic interventions of betulin, its molecular mechanisms of action from preclinical studies, and available clinical evidence from randomized controlled trials.

## 2. Natural Sources of Betulin

Historically, betulin can be traced back to 1788, when it was first observed by Lowitz [8], but its name was first coined by Mason in 1831 [9]. Betulin is a lupane-type triterpenoid with two hydroxyl groups at the 3α and 28 positions (Figure 1). One of the hydroxyl groups is a primary OH group at carbon 28, and the other is a secondary OH group at carbon 3. An alkene moiety was also observed at carbon 20. The hydroxyl and alkene groups serve as binding sites for simple modifications, whereas the pentacyclic lupane skeleton contributes to the lipophilic nature of betulin, resulting in poor aqueous solubility. Betulin is isolated from the birch cortex by sublimation or solvent extraction, and the content of betulin in the extract depends on the birch species and the part of the tree. It is found in various plant sources; however, its most abundant source is the *Betula* spp. Even so, betulin can be obtained from other non-plant sources, including mushrooms. Table 1 lists various sources of betulin, their extractants, and isolation methods.

### Methods of Isolation of Betulin

Birch bark is the primary source of betulin and offers a significant advantage for the isolation of betulin compared to other sources because of the large amount of betulin that can be obtained from the outer bark (20–30%) of birch trees as well as the large amount of bark produced from industrial waste in the paper and pulp industry. Given the growing interest in betulin, its derivatives, and their biological activities, the efficiency of the extraction procedures used to isolate betulin is crucial. Numerous studies have been published on isolating betulin from different sources [10,11,12,13,14,15,16,17,18,19,20,21,22,23,24,25,26,27,28,29,30,31,32,33,34,35,36,37,38,39]. Many of the methods employed are solvent extraction of different plant parts (Table 1) with solvents such as ethanol, methanol, chloroform, DES, hexane, water, dichloromethane, and others, with techniques of extraction involving percolation, maceration, enfleurage, and Soxhlet extraction; however, the yield generated is often quite low. Hence, the adoption of other techniques, such as supercritical CO_2_ extraction, microwave-assisted extraction, sonication, and pressurized liquids, while column chromatography and recrystallization were employed for the purification of betulin [16,20,21]. According to Šiman et al., betulin of high purity (>99%) can be obtained from other compounds by a five-step method that consists of the first removal of acids and impurities with calcium hydroxide, benzene extraction to remove lupeol, recrystallization from ethanol, removal of residual impurities on silica gel, and final recrystallization from ethanol [3].

## 3. Pharmacological Activities of Betulin

### 3.1. Protective Effects of Betulin on Cardiovascular Diseases

Cardiovascular disease (CVD) is a group of conditions with several root causes that involve the heart and blood vessels, such as heart failure, cardiomyopathy, arrhythmia, ischemic heart disease, congenital heart disease, etc.; however, the underlying mechanisms vary depending on the disease. An estimated 17.9 million people died of cardiovascular disease in 2016, accounting for 31% of all deaths worldwide and the largest cause of death globally. According to the American Heart Association, cardiomyopathy is a heterogeneous group of cardiac muscle diseases, usually with inappropriate ventricular hypertrophy or dilation, with varying causes and can either be genetic or acquired, i.e., develop from other disease conditions [40].

It was found that betulin inhibited SREBP activation by significantly decreasing endoplasmic reticulum stress markers (BIP, CHOP, and PDI) in murine H9c2 cardiomyoblast cells, markedly improving cardiac morphological characteristics and alleviating pathological cardiac conditions (such as degenerating muscle fibers, vasculitis and infiltrating immune cells); lowering cardiac lipid levels (acetyl CoA carboxylase (ACC), FAS, and LDL); and increasing cardiac levels of ABCA1 [41].

A recent study using C57BL/KsJ-*db*/*db* mice and H9c2 cells discovered that betulin significantly decreased the ST-segment of the electrocardiogram and the area of myocardial infarction; improved myocardial function and cardiac pathological changes; upregulated Sirt1 expression while downregulating ASC, IL-1β, caspase-1, NLRP3, p-NFκB, CD68, and Gr-1 [42,43]. Moreover, oral betulin treatment (30 mg/kg per day for 14 weeks) to LDLR-knockout mice resulted in decreased lesions in the aortic arch and thoracic aorta and increased stability of atherosclerotic plaques [44]. Atherosclerosis is a major contributor to cardiovascular disease, and in HFD-apoE^−/−^ mice, betulin (20 and 40 mg/kg) inhibited atherosclerotic lesions and enhanced cholesterol efflux by overexpressing the levels of ATP-binding cassette transporters, ABCA1 and ABCG1. The authors further showed that betulin-enhanced ABCA1 expression in THP-1 and RAW264.7 cells was mediated by repression of SREBPs and inhibition of its target genes (HMG-CR, FAS, LDLR) [45]. 

### 3.2. Protective Effects of Betulin on Diabetes

About 537 million people globally are living with diabetes which is expected to increase to about 783 million in 2045 (www.idf.org; accessed on 22 May 2023). Diabetes mellitus is a long-term metabolic disorder featuring hyperglycemia, hyperlipidemia, and dysfunctional insulin secretion [43] The disease condition is accompanied by severe and debilitating comorbidities, including microvascular diseases: diabetic nephropathy, neuropathy, and retinopathy, as well as macrovascular diseases, such as coronary heart disease and peripheral vascular diseases. Triterpenoids, particularly the lupane-type, may be a promising therapeutic drug for diabetes because of their diverse biological actions, which include effects on glucose uptake and absorption, diabetic vascular dysfunction, and insulin secretion [46].

According to Wen et al., betulin administered to male C57BL/KsJ-*db*/*db* mice for 12 weeks at 20 and 40 mg/kg doses significantly decreased blood sugar, serum insulin, total triglyceride, and total cholesterol levels [43]. Other researchers have demonstrated that betulin restores insulin resistance by improving glucose tolerance, inhibiting lipid peroxidation in the hippocampus, modifying basal learning performance, reducing inflammatory cytokines (IL-6, IL-1β, TNFα), and inhibiting NFκB signaling axis in diabetic rats [47]. In a different investigation, betulin potentiated insulin-stimulated glucose absorption by increasing PPAR-γ activity in 3T3-L1 adipocytes [22] and significantly decreased glucose levels time-dependently in healthy and Alloxan-induced diabetic rabbits (0.2 g/kg), hence, exhibiting hypoglycemic effects [48]. Additionally, an *in silico* study using betulin isolated from *Ruellia tuberosa* L. was shown to be a non-competitive α-amylase inhibitor [49,50].

Several other authors have corroborated the excellent inhibitory activity of betulin on α-amylase and α-glucosidase [34,51,52,53]. Inhibitors of alpha-amylase and alpha-glucosidase have been therapeutically shown to improve post-prandial hyperglycemia in diabetic patients by delaying the rate of glucose metabolism [51,54] In another study, C57BL/6J mice fed a high-fat diet demonstrated that betulin treatment improved insulin sensitivity and glucose tolerance. The work further reported that betulin inhibited SREBP expression, downregulated SREBP-2 target genes (FAS, ACC, and SREBP-1c), and significantly increased adiponectin, LPL, and PPAR-γ expression in white adipose tissue, where the overexpression of these genes was thought to be antidiabetic and anti-inflammatory [44]. Impaired wound healing is a major risk factor associated with diabetes mellitus. An *in vitro* model of fibroblasts and keratinocytes obtained from both diabetic and non-diabetic donors evaluated for betulin-enhancing wound-healing effects led to enhanced mRNA levels of proinflammatory cytokines, chemokines, and mediators crucial for wound healing such as IL-6, TNF, IL-8 and RANTES [55]. Betulin demonstrated a range of advantageous benefits as an SREBP inhibitor in different experimental models, indicating betulin could be a promising therapeutic target to treat metabolic illnesses, particularly diabetes mellitus, and atherosclerosis.

### 3.3. Protective Effects of Betulin on Cancer

Nowadays, various therapies are employed clinically to improve cancer, including drugs or drug combinations, such as cisplatin, doxorubicin, etoposide, temozolomide, 5-fluorouracil, gefitinib, sorafenib; however, these drugs have various undesirable effects that greatly limit their applications (https://www.cancerresearchuk.org/about-cancer/treatment/chemotherapy/side-effects; https://www.cancerresearchuk.org/about-cancer/cancer-in-general/treatment/cancer-drugs/drugs (accessed on 22 May 2023)). Hence, the need to discover new anti-cancer drugs or drug combinations with less toxicity and side effects. Understanding the association between naturally occurring bioactive compounds and known cellular targets is critical for developing effective cancer therapy strategies. There exists a considerable body of literature on many natural substances inducing intrinsic (mitochondrial) and extrinsic (Fas/FasL) apoptosis in cancer cells [56,57,58,59]. Other mechanisms of action are demonstrated by downregulating the expression of angiogenic and metastatic proteins (matrix metalloproteinases, MMPs, and VEGF) and by inhibiting several inflammatory mediators, including IL-6, iNOS, IL-8, COX2, IFN-γ, and TNF-α [60,61,62,63,64].

Natural substances or secondary metabolites widely dispersed in various organisms can exhibit anticancer effects or enhance the effectiveness of common chemotherapies. Studies on the anticancer activities of betulin are well documented (Table 2); it is also well acknowledged that betulin affected these activities through several mechanisms such as: (1) causing mitochondrial damage that results in cytochrome c release and apoptosis induction, (2) inducing apoptosis via caspase 9 and 3 activation pathway, (3) induction of autophagy, (4) over-expressing of PKC-δ, (5) inducing the death receptors via caspase 8 activity, (6) cell cycle arrest.

Numerous investigations have corroborated that betulin was effective in inhibiting the growth of prostate, breast, colorectal, and lung cancer cell lines [65,66]. A study on the human cell lines: epidermoid carcinoma (A431), cervix cancer (HeLa), and breast adenocarcinoma (MCF-7) demonstrated that betulin (13.28 µg/mL) extracted from *Betula pendula* exhibited 81.39, 70.30, and 35.54% inhibition, respectively [18]. In another *in vitro* research by Dehelean and co., betulin inhibited the growth of cancer cell lines in a dose-dependent manner (IC_50_ values: HeLa, 6.67 µM; A431, 6.76 µM; MCF7, 8.32 µM) by exhibiting a gradual nuclear condensation, fragmentation, and contraction, characteristics of cell apoptosis. For further confirmation, they performed an *in vivo* study using a chick chorioallantoic membrane, in which betulin demonstrated anti-angiogenic activity by reducing newly generated capillaries, particularly in the mesenchyme, without modification to the stromal architecture [67]. Additionally, a study on human gastric cancer (SGC7901 cells) revealed that betulin prevented cell proliferation and clonogenic growth of gastric cancer cells via activation of intrinsic apoptotic signaling axis by downregulating anti-apoptosis proteins XIAP and Bcl-2 [68].

It was also discovered that betulin significantly inhibited the viability of several human cell lines, including cervix cancer (HeLa), lung cancer (A549), liver cancer (HepG2), and breast cancer (MCF-7) with IC_50_ values ranging from 10–15 µg/mL and exhibited moderate antitumor activity in hepatoma (SK-HEP-1), prostate cancer (PC-3), and lung cancer (NCI-H460) with IC_50_ values of 20–60 µg/mL. The study further revealed that betulin induced apoptosis by activating caspase 9 and 3/7 but not caspase 8 in HeLa cells [69]. Mullauer et al. evaluated the effects of betulinic acid and betulin in combination with cholesterol on Jurkat T leukemia cells and described that the combination of betulin and cholesterol was effective in killing cancer cells *in vitro* [70]. Mitochondrial damage is responsible for betulin-cholesterol-induced apoptosis in Jurkat cells, according to the authors. This damage leads to apoptosis and the release of cytochrome c, which is completely independent of Bcl-2 [70]. Another study on the antiproliferative effects of betulin on several cancer cells showed that betulin inhibited the growth of nervous system tumor cells (SK-N-AS, C6, and TE671), peripheral tissues (HT-29, T47D, FTC238, and A549), blood malignancies (RPMI8226, and Jurkat IE.6) and primary culture (HPOC, HPCC, and HPGBM). Additionally, betulin effectively reduced the migration of glioma (C6), lung cancer (A549), and medulloblastoma (TE671) cells and considerably caused apoptotic cell death in A549 cells at a low dose of 5 µM [71]. This raises the possibility that betulin can be used as a chemopreventive agent for patients with a higher risk of developing metastases in lung cancer, given that a non-toxic concentration of betulin can substantially inhibit the migration of multiple tumor cells and that the same dose can significantly inhibit the proliferation of tumor cells [71].

Oral administration of betulin abated lung metastasis of CT26 cells in Balb/c mice via cell cycle arrest, autophagy, and apoptosis through the regulation of the AMPK, PI3K/Akt/mTOR, and MAPK signaling pathways [72]. Other studies have also demonstrated that betulin nanoemulsion has a relative anti-angiogenic effect, low cytotoxicity, and inhibition of VEGF expression at the chorioallantoic membrane vascular level in chick embryos and demonstrated inhibition of skin tumor appearance and promotion by histological findings [73]. With an IC_50_ of 8 µM, betulin considerably slowed the growth of SK-N-SH cells in a more recent investigation on neuroblastoma. Additionally, it increased PKC-δ activity, which in turn activated caspases 3, 8, and 9, triggering endogenous apoptotic pathways in SK-N-SH cells that are mediated by mitochondria [74]. Similarly, betulin showed chemopreventive effects against cadmium-induced cytotoxicity in HepG2 cells. Betulin prevents apoptotic processes by inhibiting ROS production, cadmium-induced upregulation of Fas, caspase-8-dependent Bid activation, and subsequent inhibition of the mitochondrial pathway [75].

Given the number of studies conducted on betulin as a chemotherapeutic agent, some other studies have considered the use of betulin as a combination therapy. Co-treatment of human renal carcinoma cells (RCC4) with betulin (10 μM) and etoposide (10 μM) synergistically increases the levels of cleaved PARP and decreases MDR1 [76]. In another study, a combination treatment of betulin and cisplatin caused 50% inhibition of H460 cells at concentrations less than 5 μM as compared to the individual drugs [27] Likewise, another study on the co-treatment of hepatocellular carcinoma tumors with betulin and sorafenib revealed that betulin prevented the resistance of HCC cells to sorafenib [77]. Despite all of these recent positive studies on the chemotherapeutic properties of betulin, earlier studies on the cytotoxic effects of betulin have shown that it has no or limited cytotoxic effects on cancer cell lines [78,79].

**Table 2:** Molecular mechanisms of betulin in tumor cells in different preclinical studies.

Experimental Model		Dose/Concentration	Pharmacological Indicator	Molecular Mechanism	References
Gastric SGC7901 cells	-	IC_50_ 13 µg/mL	ROS ↑ Caspase 3 ↑ cleaved PARP ↑ Smac ↑ cytochrome c ↑ Bax ↑ Bak ↑ Bcl-2 ↓ XIAP ↓ Caspase 9 ↑ Bcl-xL * c-IAP1 * c-IAP2 *	Mitochondrial pathway	[68]
Human hepatoma HeLa cells	-	IC_50_ 10 µg/mL	caspase9 ↑ caspase3/7 ↑ caspase 8 * cytochrome c ↑ Smac ↑ Bax ↑ Bak ↑	Mitochondria pathway	[69]
Human lung adenocarcinomaA549 cells	-	20 µM	enoyl-CoA hydratase ↓ PCBP 1 ↓ isoform 1 of 3-hydroxyacyl-CoA dehydrogenase type 2 ↓ malate dehydrogenase ↑ HSP 90-alpha 2 ↓ aconitate hydratase ↑ arginine/serine-rich splicing factor 1 ↑	None	[65]
HepG2 cells	-	10 µg/mL	Caspase 3 ↑ Caspase 9 ↑	None	[80]
Murine CT26 human HCT116	BALB/c mice injected intravenously with CT26 cells	0–8 μM 5 and 10 mg/kg for 14 days	Bcl-2 ↓ CyclinD1/CDK4 ↓ Bax ↑ cleaved caspase-3, -9, and -PARP ↑ LC3-II ↑ beclin ↑ p-ERK ↓ p-p38 ↓ Bcl-xL ↓ p-JNK ↓	AMPK activation Blockage of the MAPK signaling pathway Inhibition of Pi3k/Akt/mTOR signaling pathway	[72]
-	Female rats DMBA (25 mg/kg b.wt. s.c injection)	20 mg/kg/b.wt. in corn oil (1 mL)	TBARS ↓ LOOH ↓ CAT ↑ SOD ↑ GPx ↑ Vit C ↑ Vit E ↑ GSH ↑ AhR ↓ ARnT ↓ CYP1A1 ↓ Keap1 ↓ HO-1 ↑	Inhibition of MAPK proteins Activation of AhR/Nrf2 signaling axis	[81]
Human renal carcinoma cells (RCC4)	-	10 and 25 μM	cleaved caspase3/7 ↑ cleaved caspase 8 ↑ cleaved PARP ↑ TRAIL R1/DR4 and R2/DR5 ↑ TNFR1 ↑ Bax ↑ XIAP ↓ PUMA ↑ Bcl-2 ↓ cleaved caspase 9 ↑	Activated mitochondrial apoptotic signaling and inhibited NFκB pathway	[76]
Non-small lung cancer cells (H460)	-	11 and 30 μM	p53 ↓ Bcl-2L1 ↓ MMP2/9 ↓ BAK ↑ BAX ↑ caspase 3 ↑ caspase 6 ↑ caspase 9 ↑ caspase 8 ↓ HRK ↑ VEGF ↓ COX2 ↓ osteopontin ↓	Mitochondrial intrinsic pathway	[27]
Human colon cancer cells (HCT116 and HT29)	-	10 μg/mL	cleaved caspase 9 ↑ cleaved caspase 3 ↑ cytochrome c ↑ Bim ↑	Induction of NOXA	[82]
Renal cell carcinoma (786-O and Caki-2)	-	5 μM	p-S6 ↓ p-4EBP1 ↓ PKM2 ↓ HK2 ↓	Modulation of mTOR signaling pathway	[83]
Human osteosarcoma cell (HOS and MG-63)	-	0–20 μM	cleaved caspase 3 ↑ cleaved PARP ↑ p-mTOR ↓ cleaved caspase 9 ↑ p-4E-BP1 ↓ LC3-II ↑ cleaved caspase 8 ↑ p-Akt ↑	Inhibition of mTOR signaling Activating autophagy	[84]
-	Male Wistar Rat (DMH 20 mg/kg b.wt. s.c.)	20 mg/kg b.wt for 16 weeks	GSH ↑ GPx ↑ SOD ↑ CAT ↑ IL-1β ↓ CYP450 ↓ CYT-b5 ↓ GST ↑ GR ↑ COX-2 ↓ iNOS ↓ TNF-α ↓ PCNA ↓ cyclin D1 ↓ IL-6 ↓	None	[85]
human ovarian carcinoma cells (OVCAR-3)	-	0–120 μM	Cyclin-D1 ↓ Bad ↑ Bax ↑ Bcl-2 ↓ Bcl-xL ↓ Cyclin-B1 ↑ Cyclin-E1 ↑	modulating mTOR/Pi3k/Akt signaling pathway	[86]

N.B: ↑—upregulate/increase, ↓—downregulate/decrease, * signifies no change poly(rC)-binding protein 1 (PCBP-1), heat shock protein 90-alpha 2.

Furthermore, betulin was shown to inhibit the expression of some inflammatory factors that augment and sustain a tumorigenic environment, including IL-6 and TNF-α, in the colon tissues of experimental and control animals [85]. Other studies further demonstrated that betulin treatment inactivated the activity of protein kinase B (Akt)/mTOR signaling, which enhances other proapoptotic mediators [72,83,84,86].

### 3.4. Protective Effects of Betulin on Liver Diseases

There are hundreds of liver diseases caused by viruses, toxins, genetic factors, alcohol, obesity, unknown causes, and other factors. Liver damage is a clinical manifestation that poses a life-threatening risk associated with a high mortality rate. Chronic diseases such as diabetes can also progressively affect the liver, thereby impairing liver function. Ample evidence has shown the beneficial effect of betulin on hepatic damage, with its spectrum of activities ranging from inhibition of SREBP-1 to upregulation of Sirt1, to reduction of NFκB signaling, etc.

Since sustained excessive alcohol use can deteriorate the liver’s condition, researchers have modeled this disease pathology by inducing steatohepatitis via ethanol administration. Previous work showed that betulin (100 mg/kg b.wt.) attenuated steatosis, inflammation, and fibrosis in the liver of rats fed chronically with ethanol. The study also showed that betulin treatment could significantly improve histopathological signs of steatohepatitis, reduce liver function markers (AST and ALT) and liver and serum triglyceride levels, and restore redox imbalance by increasing GSH content and decreasing superoxide anions and TBARs concentration [87,88]. Similarly, another study demonstrated that betulin exerts hepatoprotective effects on alcohol-induced liver damage in mice through the Sirt1/LKB1/AMPK signaling pathway by inhibiting ethanol-induced activation of SREBP-1 and NFκB [89]. Szuster-Ciesielska et al. reported that betulin treatment inhibited the production of superoxide anions, procollagen type I, MMP-2, TIMP-1, and -2, α-SMA, and cells’ motility in acetaldehyde-induced toxicity in rat hepatic stellate cells (CFSC-2G cells) [90].

Furthermore, another study revealed that betulin decreased serum levels of ALT, AST, and triglycerides; inhibited EtOH-induced acidophilic necrosis; ameliorated liver histopathological changes; significantly decreased activity of CYP2E1 and expression of SREBP-1; markedly lessened the expression of TLR4; and improved phosphorylation of STAT3 *in vitro* and *in vivo* [91]. Elsewhere, betulin, betulinic acid, and oleanolic acid (1 μM each) were investigated for their hepatoprotective activity against ethanol-induced cytotoxicity in HepG2 cells, with betulin being the most active protectant of HepG2 cells against ethanol-induced cytotoxicity [92]. Another study proposed that betulin limited fibrosis formation and attenuated cisplatin-stimulated hepatic damage in rats by targeting apoptosis and NEK7-independent NLRP3 inflammasome pathways [93,94]. In addition, betulin mitigated increased levels of inflammatory factors, including IL-1α, IL-1β, IL-6, IL-18, TNF-α, TGF-β, and MMP2 in ethanol-induced liver stellate cells and chronic EtOH-treated rodents [87,88,94,95]. The hepatoprotective effects of betulin as well as its potential molecular pathways are briefly elucidated in Table 3, showing the concentration and dose of administration of betulin.

### 3.5. Protective Effects of Betulin on Inflammation 

Inflammation is a part of the host’s front line of defense against injury and infections. It is a biological process that results from the disruption of tissue homeostasis due to a variety of physical, chemical, or biological factors, including toxins, alcohol, pathogens, and so on [6] Inflammation is a complex, dynamic process regulated by multiple signaling pathways. It requires the interaction of different cells and modulates a wide range of cellular responses and homeostasis [7]. It is an adaptive response to noxious stimuli, such as tissue damage. Inflammation is divided into acute and chronic inflammation, where acute is beneficial to the host and is dominated by neutrophil infiltration characterized by classical signs of redness, heat, and swelling. However, when inflammation continues for a long time, mainly involving cellular infiltration of macrophages and lymphocytes, it becomes chronic, leading to the development of different chronic diseases [6].

The inflammatory pathway includes four parts: inducers, sensors, mediators, and effectors. Inflammatory inducers such as PAMPs, allergens, and AGEs trigger the production of multiple inflammatory mediators that change the functionality of the inflammatory effectors (organs and tissues) [6]. Several groups of inflammatory mediators can be produced during an inflammatory response, among which chemokines, lipid mediators (platelet-activating factors and eicosanoids), and cytokines have been extensively studied.

As inflammation is a hallmark of many chronic diseases, there is a need for natural compounds that can effectively inhibit inflammation and target multiple disease-related signaling pathways. Traditionally, drugs that block inflammatory mediators and inhibit eicosanoid biosynthesis have been used to treat inflammation; however, targeting multiple targets rather than a single target is preferable in complex diseases such as inflammation. Furthermore, exploring the anti-inflammatory potential of natural products is a safer option in terms of therapeutic efficacy, side effects, and negative compensatory mechanisms [96].

#### 3.5.1. Preclinical Evidence

Earlier research conducted to investigate the effects of betulin on inflammatory damage induced by *S. aureus*-mastitis in female BALB/c mice showed that betulin ameliorated histopathological changes and suppressed the expression of IL-1β, TNF-α, and IL-6 initiated by the inflammatory injury, acting as a protective anti-inflammatory agent against mastitis [97]. Additionally, betulin prevented AML-12 or RAW 264.7 cells’ activation of P2X7r-NLRP3 in response to EtOH- or lipopolysaccharide-induced inflammation [94]. Sepsis is a complex disease that is established in various modes, including lymphangitis, encephalopathy, renal failure, heart failure, and liver injury, all of which arise from an inflammatory response [5]. A study conducted on cecal ligation and puncture (CLP)-induced sepsis revealed that intraperitoneal administration of betulin (4 or 8 mg/kg) boosted the survival rate of sick rats by 80 and 60% at 48 and 96 h compared to the negative control. Additionally, betulin administration attenuated histopathological alterations, ALT and AST levels, serum TNF-α, IL-1β, and IL-6 levels, and HMGB-1 (high mobility group box 1) levels, blocked the production of phosphorylated NFκB/p65, and upregulated IκBα in septic rats [5].

Remarkably, a similar protective effect of betulin was observed in a study conducted to assess the renal-protective effect of betulin. Zhao et al. set out to demonstrate the reno-protective benefits of betulin therapy *in vitro* and *in vivo* because prior research has shown the significance of excessive inflammatory cytokines in the pathophysiology of organ failure in sepsis [98]. The results of the study showed that betulin significantly reduced the concentrations of creatinine and BUN, renal tubular damage, and secretion of pro-inflammatory cytokines (TNF-α, IL-6, and IL-1β) in the kidneys of septic rats and LPS-induced glomerular mesangial cells. TLR4 and HMGB-1 mRNA and protein expression levels in the kidneys of infected rats are also downregulated by betulin at the same time and suppress the activation of NFκB signaling pathways. Additionally, Pfarr et al. discovered that although having no effect on IL-12p70, betulin had a unique immunostimulatory effect on BMDCs via boosting IL-12p35 mRNA expression [99]. This causes T-lymphocytes to be stimulated, which can be seen by the increased production of IL-2 and IFN-γ by cytotoxic T cells in spleen cell coculture assays. Notably, betulin inhibited rabbit articular chondrocytes exposed to interleukin-1β, downregulated the expression of matrix metalloproteinase-1, -3, and -13, ADAMTS-4 and -5, and upregulated type II collagen production as well as MMP-3 protein *in vivo* [100]. The effects of betulin on inflammatory mediators are summarized in Table 4.

#### 3.5.2. Molecular Mechanisms: Multiple Targets of Betulin 

Betulin may exert its anti-inflammatory effects via a multi-target mechanism, including inhibition of ROS production, inhibition of TNF-α, inhibition of pro-inflammatory cytokines, activation of the Nrf2-associated signaling pathway, post-transcriptional inhibition of iNOS, inhibition of the NFκB pathway, upregulation of PPAR-γ expression, and modulation of the STAT3 signaling pathway.

- Modulation of inflammatory cytokines

Inflammatory cytokines are produced in response to invading pathogens and are primarily released from immune cells to recruit leukocytes to the site of injury or infection. They include interleukins (IL), interferons (IFN), chemokines, tumor necrosis factors (TNF), and so on. There are pro and anti-inflammatory cytokines, where proinflammatory mediators facilitate, and anti-inflammatory cytokines inhibit inflammation. Through a complex web of interactions, cytokines modulate the immune system. However, excessive synthesis of inflammatory cytokines can result in organ failure, tissue damage, hemodynamic abnormalities, and, finally, death [7].

Betulin inhibited overexpressed levels of proinflammatory cytokines (TNF-α, IL-6, IL-1β) in experimentally induced septic rats, which are indicative of early sepsis [5]. Other studies demonstrated that betulin decreased levels of pro-inflammatory mediators, such as tumor necrosis factor-α (TNF-α), matrix metalloproteinases (MMP-2 and 9), and interleukins (IL-1β, IL-2, IL-4, IL-5, IL-6, IL-13, and IL-17) [26,102,104,105,106,107]. Contrary to these results, betulin was shown to increase proinflammatory cytokines in primary keratinocytes and ex-vivo porcine wound healing model and re-epithelization. According to the authors, betulin significantly upregulated RANTES, TNF-α, IFNγ, MIP-1α and β, and IP-10, all of which are proinflammatory mediators involved in the inflammatory phase of the wound healing process and enhanced migration of keratinocytes, which is essential for the second phase of wound healing [103].

- Inhibition of reactive oxygen species (ROS) production

ROS induces chronic inflammation by inducing pro-inflammatory mediators such as COX2, IL-6, TNF-α, NFκB, IL-8, etc., and can also combine with nitric oxide to form reactive nitrogen species, which can induce nitrosative stress that can add to the pro-inflammatory burden of reactive oxygen species. Thus, to defend against reactive oxygen species, cells are equipped with strong antioxidant enzymes: glutathione reductase, superoxide dismutase, catalase, glutathione peroxidase, and heme oxygenase; and non-enzyme defenses: glutathione, thioredoxin, and melatonin, where, superoxide dismutase and catalase are the body’s major antioxidant defenses against reactive oxygen species [108]. In fact, preclinical trials have shown that treatment with compounds that act as antioxidant chelators, detoxification molecules, or antioxidant enzymes can prevent ROS-mediated damage [109].

When betulin (10 M) was used on HepG2 cells, the amount of superoxide anion and hydrogen peroxide generated by acetaminophen and ethanol-induced ROS considerably reduced [110]. Also, by preventing tyrosyl phosphorylation, betulin repressed superoxide production in human neutrophils [10]. Bai et al. also found that betulin had antioxidant properties by scavenging DPPH and hydroxyl radicals [20]. Similar to this, betulin showed inhibitory activity (IC_50_ 6.88 μg/mL) against H_2_O_2_-induced cytotoxicity in PC12 cells [11]. In ovalbumin-induced asthmatic mice (female BALB/c mice), betulin inhibited the proliferation of inflammatory cells, decreased ROS production and levels of oxidative stress markers, increased antioxidant enzymes, attenuated levels of pro-inflammatory cytokines, increased levels of interferon-gamma (IFN-γ), as well as downregulated expressions of tissue transglutaminase (tTG), TGF-β1 and MMP-9 [102]. Some authors have further demonstrated that betulin affects the viability of human renal proximal tubular epithelial cells (RPTECs) by reducing CAT and MDA levels and increasing the enzyme activity of GPx and SOD, thereby affecting the antioxidant system [111].

- Action on the Nrf2 signaling pathway

Nrf2 is a member of the basic leucine zipper transcription (bZIP) factor of the cap ‘n’ collar family that plays a crucial role in regulating cellular homeostasis. Nrf2 regulates the expression of antioxidant proteins, protects against oxidative damage by negative stimuli, and exerts pleiotropic effects in regulating metabolic processes, mitochondrial physiology, inflammation, autophagy, immune responses, cancer prevention, treatment, etc. [112]. Nrf2 regulates the expression of phase II cytoprotective enzymes such as HO-1, SOD, GST, NQO1 (NAD[P]H: quinone oxidoreductase-1), and -GCL (-glutamyl cysteine ligase), as well as biological processes like cell development, differentiation, apoptosis, proliferation, and hematopoiesis [113]. Nrf2 is regulated at several levels by various factors, including at transcriptional levels (NFκB, AhR-ARNT, ATF4, etc.), post-transcriptional levels (miRNA, RBPs, alternative splicing), post-translational levels (ERK, JNK, p38, PERK, CK2, GSk3, PKC), as well as regulators of Nrf2 stability (keap1, βTrCP, HRD1, WDR23, CRIF1) [112]. Studies have also revealed that, in addition to keap-1, upstream kinases like protein kinase C, MAPKs, protein kinase RNA-like endoplasmic reticulum kinase (PERK), and phosphatidylinositol-3-kinase/Akt trigger the phosphorylation and subsequent translocation of Nrf2, which in turn triggers an antioxidant cascade [109,114].

Nrf2 activation is associated with protection against different disease conditions, including cancer, inflammation, CVDs, acute and chronic lung injury, and neurodegenerative and autoimmune diseases [115]. A key therapeutic intervention for several pathological conditions is the discovery of small molecules that can modulate Nrf2. It is necessary to demonstrate how betulin regulates different signaling pathways at protein and transcriptional levels to understand betulin’s mechanism of action in exhibiting anti-inflammatory activities. Multiple studies have been conducted using different experimental models to investigate the modulatory activity of betulin on the Nrf2 signaling pathways. The results imply that betulin has protective effects in different disease conditions by modulating the Nrf2 signaling pathway and its downstream genes [59,109,116].

**Figure 3:**
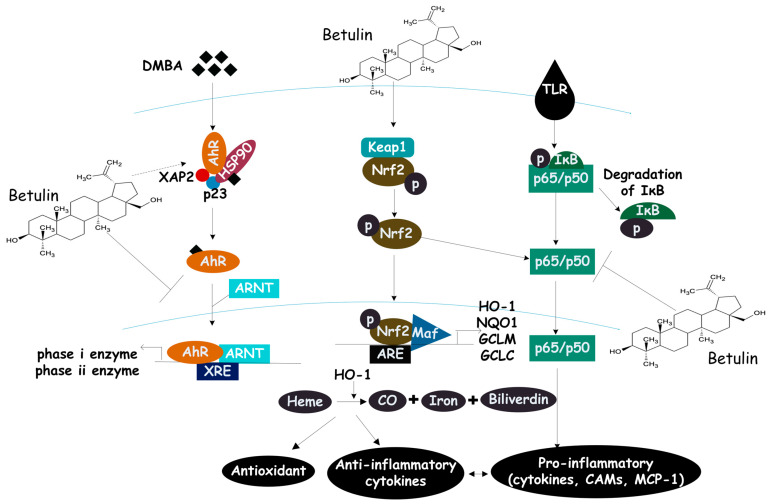
How betulin modulates the HO-1/Nrf2 signaling pathway. The antioxidant response elements (ARE) and Maf proteins work together to create a heterodimer with Nrf2 after betulin releases it from the Keap1:Nrf2 complex. This promotes the transcription of antioxidant genes while inhibiting the AhR signaling axis. By promoting the production of phase II cytoprotective enzymes and heme oxygenase-1, betulin exerts cellular antioxidant effects.

To this end, betulin administration increased the protein expression of HO-1 and Nrf2 in the hippocampus and serum of STZ-induced diabetic rats compared with diabetic controls and prevented cognitive decline in diabetic rats [47]. Arivazhagan & Subramanian claimed that AhR/Nrf2 signaling plays a major role in the treatment and prevention of chemically induced oxidative tissue damage [117]. Oral administration of betulin exhibited protective activity against DMBA (7,12-dimethylbenzanthracene)-induced mammary cancer by decreasing the expression of AhR, ARnT, CYP1A1, and keap1 while augmenting HO-1 and Nrf2 in the mammary tissues of tumor-bearing rats [81]. In another study, oral treatment with betulin abolished DMH-induced colon carcinogenesis in rats by significantly reducing phase I enzymes (Cyp 450 and Cyt-b5) and increasing phase II detoxification enzymes (GST and GT) [85]. Although there may be multiple mechanisms regulating each phase II gene, the Keap1-Nrf2-ARE signaling pathway unites most phase II genes and are important for cancer prevention. An *in vitro* study has also shown that betulin derived from *Betula platyphylla* protected HT-22 hippocampus neuronal cells against TG-induced death by reducing the production of reactive oxygen species and increasing the expression of HO-1 [116]. In addition, Ci and co-authors found that betulin induced the expression of NQO1, HO-1, and Nrf2 proteins in murine macrophages from Nrf2^+/+^ (wild-type) mice. However, the expression of these proteins was significantly repressed in cells from knock-out (Nrf^−/−^) mice. They further showed that betulin led to a dose- and time-dependent increase in Nrf2-targeted antioxidants and detox proteins: NADPH, GCLC, GCLM, and HO-1 [109]. Betulin-treated chondrocytes promote HO-1 expression and Nrf2 nuclear translocation through the activation of the Nrf2 pathway [107]. Based on these studies’ results, betulin’s antioxidant effect seems to come from activating HO-1 through Nrf2, which then scavenges free radicals. This is possible because HO-1 catalyzes the conversion of heme to free iron, carbon monoxide, and biliverdin to bilirubin [118,119,120]. As a result, the end products of HO-1 activity are cytoprotective by reducing the rate of apoptosis and attenuating inflammatory response [121]. Figure 3 illustrates betulin’s possible mode of action on the Nrf2 signaling pathway.

- Action on the NFκB signaling pathway

The ubiquitous transcription factor, nuclear factor kappa-light chain enhancer (NFκB) of activated B cells controls gene expression in different cell types, including regulation of genes beyond the immune system, which has a variety of effects on both normal physiology and disease pathology [122,123]. For example, several inducers can activate NFκB, including TNF, IL-1, dsRNA, LPS, viruses, ultraviolet light, phorbol esters, and protein synthesis inhibitors. Furthermore, NFκB plays a significant role in controlling the immune system’s response to infection, and its dysregulation is associated with autoimmune and inflammatory diseases, cancer, viral infections, etc. [59,124]. In addition to this, NFκB modulates growth factors, cytokines, and effector enzymes in response to the ligation of many receptors involved in immunity, including TNFR, BAFFR, T-cell receptors (TCRs), CD40, B-cell receptors (BCRs), LTR, and the Toll/IL-1R family [123]. NFκB, characterized by the interaction between NFκB dimers, IκB modulators, and the IKK complex, is receptive to a variety of stimuli, and following-receptor specificity, several cellular outcomes are set in motion, suited to the individual signal received [125].

Given that NFκB regulates a large number of genes associated with inflammation, it is not surprising that persistent activation of NFκB has been reported in many inflammatory disorders, such as inflammatory bowel diseases, gastritis, inflammatory lung diseases, atherosclerosis, sepsis, arthritis, asthma, etc. [126,127]. This is because the downregulation of NFκB is associated with apoptosis, whereas the activation of NFκB upregulates anti-apoptotic genes, pro-inflammatory cytokines, growth factors, and adhesion molecules. Therefore, mechanisms that inhibit NFκB signaling have potential therapeutic applications in inflammatory and inflammatory-related diseases [128]. An earlier study found that betulin reduced the levels of phosphorylated NFκB and IκBα, its inhibitor while maximizing the level of total IκBα to protect hepatic stellate cells from ethanol-induced toxicity [95]. In LPS-induced macrophages and in rodents with acute lung inflammation caused by LPS, betulin reduced the expression of IL-6 and TNF-α while improving the expression of the anti-inflammatory cytokine IL-10. According to the authors, betulin inhibited proinflammatory cytokines by suppressing the phosphorylation of NFκB p65 protein and p-IκBα thus deactivating the NFκB axis [129]. Kawai & Akira and Sharif et al. also demonstrated that LPS binding to TLR4 activates NFκB, which regulates gene expression and supports macrophage-dependent immune response [130,131] What’s more, by preventing the activation of NFκB, betulin can lessen inflammation brought on by lipopolysaccharide. Interestingly, equivalent results were reproduced by El-Sherbiny and colleagues in acetic acid-induced ulcer colitis (UC). The study illustrated that betulin silenced ulcerative colitis-associated colonic inflammatory load by modulating the TLR4-NFκB signaling pathway as well as decreasing levels of colonic inflammatory cytokines such as TNF-α, IL-1β, and IL-6 [132]. Moreover, Zhang and co. have found that betulin inhibited the expression of pro-inflammatory cytokines and NFκB in human cardiac cells [133]. Consistent with these results, betulin attenuated renal injury via repression of activated NFκB in CLP-induced sepsis rats [98].

**Figure 4:**
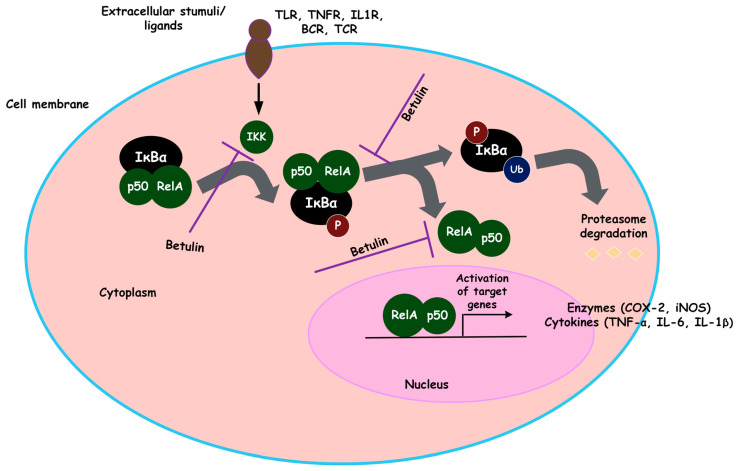
The effect of betulin on NFκB signaling axis. The activation of the NFκB signaling by cytokines results in IKK’s phosphorylation of IκBα, which causes IκBα degradation and then translocates p50/RelA to the nucleus, where it activates the transcription of NFκB targeted genes. Betulin’s inhibition of NFκB appears to be via the obstruction of IKK phosphorylation and inhibiting nuclear translocation of NFκB.

Studies have shown how betulin inhibits the NFκB signaling pathway through other related signaling pathways. Guo et al. demonstrated that the activation of PPARγ inhibits NFκB activation, which has anti-inflammatory effects [97], and a similar mechanism of action was observed in LPS/D-Gal-stimulated acute liver injury [87]. Previous studies have shown that, in addition to expressing anti-inflammatory effects by inhibiting the activation of NF-κB, PPARγ is also involved in the differentiation and activation of monocyte [134,135]. Other researchers found that betulin improved smoking-induced COPD in mice, possibly by decreasing the levels of proteins involved in the ROCK/NFκB pathway [136]. A recent study by Ren et al. concluded that via activating the AKT/Nrf2/HO-1/NFκB signaling axis, betulin moderated the inflammatory response of IL-1β-induced osteoarthritis [107]. The literature on Nrf2 clearly demonstrates its role in reducing NFκB-driven inflammatory responses in many experimental studies, possibly because both the Nrf2 and NFκB pathways control cellular redox homeostasis and responses to stress and inflammation [137,138,139]. Recent findings have also demonstrated that betulin’s ability to decrease neuroinflammation in response to LPS may be due to the multi-target inhibition of iNOS, the JNK pathway, and NFκB [140]. All these results collectively suggest that betulin can inhibit inflammation *in vitro* and *in vivo* by modulating NFκB and NFκB-associated signaling pathways (Figure 4).

- Action on the MAPK signaling pathway

The MAPK signaling pathway regulates an array of cellular processes, including proliferation, mitosis, inflammatory responses, motility, and apoptosis, while the dysregulation of these cascades is implicated in the induction and progression of different disease pathologies such as cancer, autoimmune diseases, and diabetes [141,142,143]. Soares-Silva et al. defines MAPKs as protein kinases that phosphorylate either their own dual serine and threonine residues (autophosphorylation) or those found on their substrates to switch on and off their target. In their basic form, MAPKs are catalytically inactive and only become active through multiple phosphorylations of their activation loops [144]. The MAPKs signaling cascade consists primarily of three consecutively activated protein kinases (MAP3K, MAP2K, and MAPK) and interactions with a small GTPase and/or activation by protein kinases that are downstream of cell surface receptors actuate MAP3K [145]. The MAP2K is directly phosphorylated and activated by the MAP3K, and the MAP2K then double phosphorylates the conserved tripeptide TxY (threonine-x-tyrosine) motif in the activation moiety to activate MAPK. Once activated, MAPK phosphorylates various substrates in the cytoplasm and nucleus, leading to protein activity and gene expression changes to execute appropriate biological responses [146]. Conversely, the MAPK phosphorylation cascade can be inactivated by MAPK protein phosphatase (MKP), which dephosphorylates TxY residues on MAPKs. Currently, ERK1/2 (extracellular signal-regulated kinase 1 and 2), JNK (c-Jun N-terminal kinase), p38 MAPK, and ERK5 have been recognized as the four distinct mammalian MAPK cascades. There are also other kinases that have been discovered, such as ERK3/4 and ERK7/8, whose sequences are comparable to those of MAPK cascade components [147].

ERK-1, p38, and JNK belong to a family of MAPKs that play a key role in causing oxidative stress by increasing free radicals and depleting antioxidants. These three classical pathways (ERK, JNK, and p38 MAPK) have unique sensitivities when exposed to different stimuli [148]. ERK1/2 can be activated by growth factors, hormones, and pro-inflammatory stimuli, while pro-inflammatory stimuli, cellular, and environmental stressors can also activate JNK1/2/3 and p38 [149].

Many studies have examined the therapeutic effects of betulin on inflammatory-related diseases and found that inhibition of MAPK proteins was an effective anti-inflammatory treatment [150,151,152]. One study showed that exposure of hepatic stellate cells to a clinically relevant concentration (50 mM) actuates JNK and p38. The protective role against oxidative stress and inflammation in signal transduction was proposed because betulin prevented ethanol’s induction of MAPK [95]. What’s more, betulin reduced the levels of inflammatory mediators produced by LPS, such as iNOS and COX2, as well as inflammatory signaling pathways (MAPKs) [109]. Betulin extracted from *Pyrola incarnata* possessed a neuroprotective effect by suppressing LPS-promoted phosphorylation of JNK 46/54 expressions in BV-2 microglia cells [140]. Through JNK and ERK1/2-dependent signaling pathways, betulin was also discovered to increase osteoblast development and osteoinductive actions in human osteoblasts [153]. Interestingly, betulin isolated from birch bark inhibited phosphorylation of p38 and RANKL-mediated osteoclastogenesis in bone marrow-derived macrophages, whereas p-JNK and p-ERK were not significantly altered [154]. RANKL is important for inhibiting osteoclast differentiation and proliferation, and MAPK (p38) inhibits osteoclast formation upon betulin treatment. Thus, in osteoarthritic synovial fibroblasts, betulin was found to downregulate TNF-α and IL-1β-induced inflammation by inhibiting the ERK/MEK signaling axis [155].

**Figure 5:**
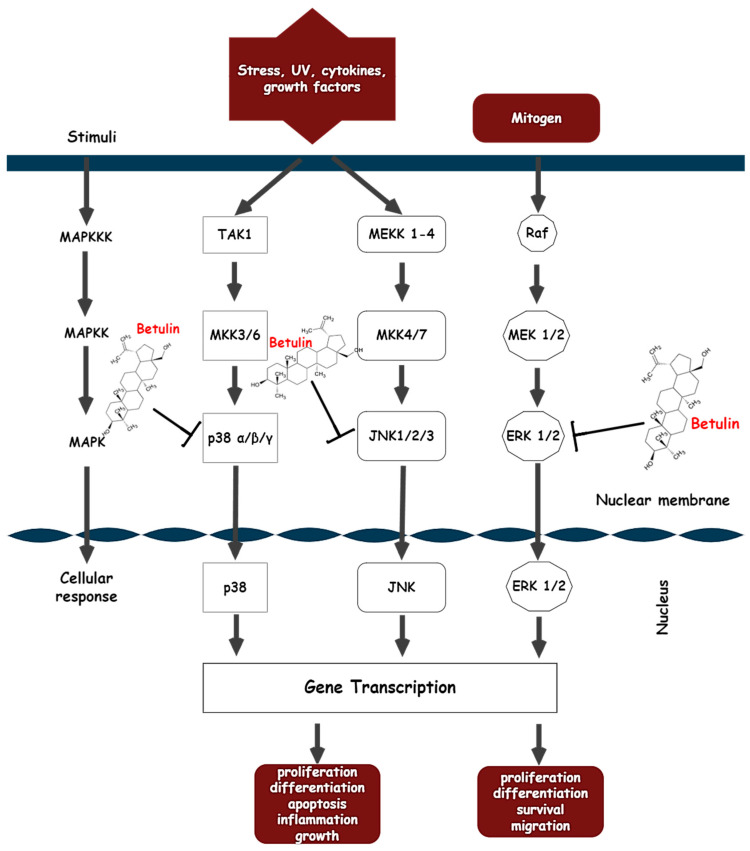
The effect of betulin on MAPK signaling pathways. Betulin blocks the overexpression of p38, JNK, and ERK in response to different stressors, thereby downregulating the transcription of MAPK-targeted genes.

MAPK signaling has been associated with cancer cell survival, and many MAPK inhibitors have therapeutic potential in the treatment of cancer by inducing apoptosis [156,157]. In different colorectal cancer cells, betulin-caspase-dependent apoptosis was mediated via the MAPK signaling pathway [72]. Hence, betulin treatment can significantly inhibit the hyperphosphorylation of ERK, JNK, and p38 in metastatic colorectal cancer cells, which may also be related to the pharmacological mechanism of betulin. Rats exposed to DMBA overexpressed JNK1, ERK1, and p38 in the mammary tissue and were repressed by treatment with betulin [81]. *Acacia auriculiformis* stem bark served as a new source for betulin synthesis, and a further *in vitro* investigation in doxorubicin-resistant chronic myeloid leukemia cells revealed that it could modulate the MAPK pathway, with activity like imatinib mesylate (a known Abelson murine leukemia viral oncogene homolog 1 kinase (ABL1K) inhibitor) [37]. The findings from these various experimental models suggest that betulin significantly inhibits inflammatory responses by deregulating the expression of MAPK proteins, further demonstrating that betulin has a significant anti-inflammatory effect and may be used as a safe and efficient natural remedy. The possible molecular mechanism of betulin on the MAPK signaling pathway is shown in Figure 5. The pathophysiologic complexity of inflammation requires a multi-target therapeutic approach, and it is likely that betulin therapy can have a place in future therapies due to its therapeutic effects and pharmacological target, making it a potential candidate, as is the case with the antiviral drug Bevirimat (a derivative of betulinic acid) [158].

## 4. Clinical Evidence of Betulin

Apart from preclinical studies, several clinical trials have also been conducted on betulin, ranging from anti-hepatitis activity to wound-healing effects as shown in Table 5 [159,160,161,162,163,164,165]. Wound healing is a complex cellular and molecular process that involves tissue regeneration and inflammation as an initial phase, whereas anti-inflammatory treatment reduces inflammation. Basically, natural products that promote the wound healing process are advantageous because wound repair involves three overlapping phases, including inflammation, new tissue formation or cell proliferation, and remodeling, and due to its complexity, many points can be targeted [103]. The first phase of wound repair is characterized by homeostasis and the initiation of a controlled inflammatory response. Various pro-inflammatory cytokines, including chemokines and growth factors, are secreted to coordinate the attraction of macrophages and granulocytes to the wound area for phagocytic and antimicrobial functions. The second phase occurs with the migration and proliferation of keratinocytes, the formation of granulation tissue and extracellular matrix by fibroblasts, as well as angiogenesis. The final remodeling phase is characterized by collagen synthesis, apoptosis, and scar formation [159]. Ebeling et al. purported that betulin enhanced the onset of the inflammatory phase of wound healing by transiently upregulating proinflammatory mediators (cytokines, chemokines, and cyclooxygenase-2); however, this temporary overexpression does not lead to a protracted inflammation [103].

According to a study on the effects of oleogel formulations on skin injuries, betulin-based oleogel significantly accelerated the healing of wounds by encouraging the migration of immortalized human keratinocytes [159]. Also, a phase II pilot clinical trial of Oleogel-S10 (which contains a betulin-rich triterpene extract) on acute and chronic wounds of 10 patients with dystrophic epidermolysis bullosa showed faster wound healing and reepithelization than in the untreated groups [160]. Other clinical studies on actinic keratoses, skin graft transplants, and burns revealed that betulin enhanced wound healing and reepithelization [162,163,166,167].

## 5. Betulin Derivatives

To better understand the structural features underlying the biological activities of betulin and to enhance its pharmacokinetics, efforts have been geared toward modifying its original carbon skeleton in order to increase its serum solubilization and broaden its range of biological activities. Recent works have focused on the synthesis of new molecules such as betulinic acid, betulone, and betulonic acids from betulin due to the ease of extraction and isolation of betulin [168]. Specifically, structural changes were mostly made at C-3 and C-28 via two methods, namely: chemical synthesis and biotransformation, with the majority of the modifications categorized into hydrolysis, isomerization, redox, condensation, carbon-carbon bond formation, and addition of functional groups that can lead to the formation of potentially pharmacologically active agents [169].

Polycyclic triterpenoids are convenient precursors for novel semi-synthetic derivatives, and there is evidence that several newly derived products with different pharmacological potentials have been obtained [2,9]. Chrobak et al. described the cytotoxic effects of newly synthesized betulin derivatives against different cell lines (breast tumor, glioblastoma, and melanoma) [170]. The alkynyl ester derivative, 29-diethoxyphosphoryl-28-propynoyloxy-lup-20E(29)-en-3β-ol was the most cytotoxic to breast tumor, glioblastoma, and melanoma cells at IC_50_ values of 0.44, 0.27, 0.38 µg/mL. According to the authors, adding a phosphonate group to the isopropenyl part of betulin made it more effective at killing tumor cells as compared to other derivatives. Another study investigated the anti-inflammatory activities of betulin and sixteen of its semi-synthetic derivatives, and findings from the study showed that a new derivative, pyrazolobetulinic acid, exhibited a wide spectrum of anti-inflammatory properties [4].

**Figure 6:**
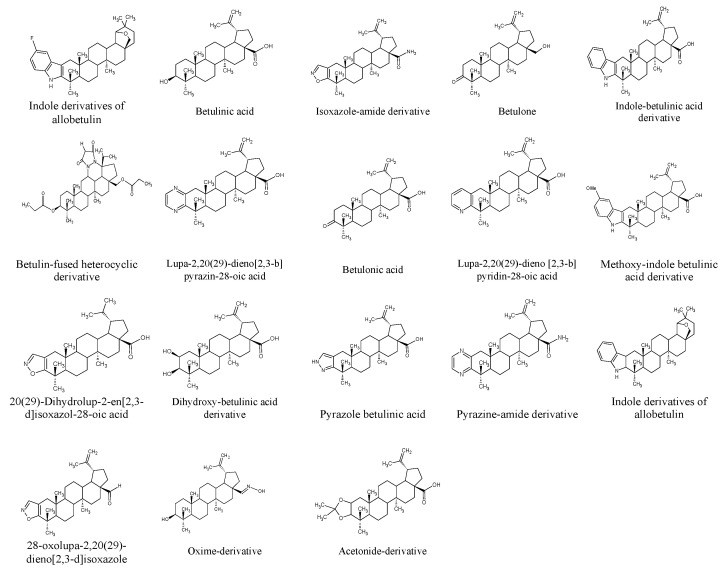
The structural formula of betulin-derivatives.

Other studies have also shown that diverse derivatives of betulin can be obtained by acetylation of betulin and its derivatives [171,172]. Furthermore, it was found that modification of betulin with GalNAc (N-acetyl-D-galactosamine) produced glucotriterpernoids, which were non-toxic *in vitro* and had a high specificity for the asialoglycoprotein receptor of hepatocytes *in silico* [173]. Elsewhere, betulone was prepared via the oxidation of betulin over synthesized Ru nanoparticle catalysts [174]. Moreover, a recent study also showed that betulin complexed with 2-hydroxypropyl-β-cyclodextrin (HPβCD) exhibited improved aqueous solubility and, hence, better bioavailability *in vivo* [88]. Figure 6 illustrates some betulin and betulinic acid derivatives.

According to Singh, biotransformation involves the use of microbial cells and enzymes to catalyze reactions, leading to compounds with relatively greater polarity [169]. When compared to chemical synthesis, its advantages include better stereo-and regioselectivity; economy; original carbon-skeleton integrity; greener synthesis; milder conditions; and simplifying reactions that are impossible with chemical methods [168]. For instance, the bioconversion of betulin at carbon position 28 synthesized betulinic acid in a one-step process but resulted in a low yield of betulinic acid [175]. Several other hydroxylated/oxygenated derivatives of betulin have also been produced through microbial transformation and are reported to possess improved polarity and pharmacological effects. A study on the conversion of betulin by *Rhodococcus rhodochrous* IEGM 66 produced a 3-oxo derivative of betulin—called betulone [176]. A similar study using a yeast strain, *Rhodotorula mucilaginosa*, on betulin, reported the production of betulone and an aromatic compound known as octadeca-11,14-dienoate [168].

Meanwhile, a fungus, *Cunninghamella blakesleeana* AS 3.910, was reported to transform betulin into betulinic acid [177]. In another study, the marine fungus *Dothideomycete* sp. HQ 316564 was also identified as the unique strain that regio-selectively catalyzed the oxidation of betulin to betulone [178]. Another fungus strain, a culture of *Mucor subtilissimus* CGMCC 3.2456, was shown to transform betulin into nine novel hydroxylated compounds. Most of these metabolites exhibited marked inhibitory activities against LPS-induced NO production in macrophages [179]. In another study, *Armillaria luteo-virens* Sacc QH, a fungus, also bio-catalyzed the transformation of betulin to betulinic acid [180]. Moreover, previous findings on betulin’s metabolic pathway using human and rat liver microsomes revealed two phase II pathways (glucuronidation and sulfonation) as its main metabolic pathways. In the rat models, UDP-glucuronosyltransferase family 1 members A3 and A4, enzymes of the glucuronidation pathway, were the major liver enzymes responsible for the possible formation of the C3-hydroxyl betulin glucuronide, while sulfotransferase 2A1 was found to be the main isoenzyme responsible for betulin sulfonation in human liver cytosol [181]. Another group of authors established 34 metabolites of betulin *in vitro* and *in vivo* using the uhplc-q-tof-ms/ms system, including 32 phase I metabolites and 2 phase II metabolites [182]. Likewise, following the oral administration of betulin, 56 phase I and 6 phase II metabolites of betulin were found utilizing the uhplc-q-tof-ms/ms system. The authors demonstrated that deoxidation, dehydration, dehydroxylation, demethylation, and conjugation with acetylcysteine, cysteine, taurine, and sulfate were the primary biotransformation routes of betulin [183].

## 6. Pharmacokinetics of Betulin

Hu et al. suggested that understanding the metabolism of a drug can assist in explaining and predicting the cascade of events associated with the potency and toxicity of the drug [184]. Studying the metabolism of betulin *in vivo* has proved challenging, especially considering the chemical makeup of betulin, its lipophilicity, and its insolubility in aqueous media. In addition, there may be discrepancies in the currently available data due to the limited number of research investigating the pharmacokinetics of betulin, low bioavailability, route of administration, and doses. Accordingly, Jäger and colleagues evaluated the blood samples of both male and female rats intraperitoneally administered with betulin (60, 180, and 540 mg/kg) for 4 h, and a significant increase in plasma betulin level was seen to be time-dependent over a period of 4 h, reaching a dose-independent serum level of 0.13 μg/mL for all dose groups. In contrast, dose-dependency was detected with subcutaneous betulin administration in dogs, with a maximum betulin plasma level of 0.33 μg/mL at 300 mg/kg after 28 days [185].

In another *in vivo* study involving the pharmacokinetics of the betulin nanosystem in rats, a single dose was administered endotracheally, resulting in the detection of a maximal concentration of betulin in the blood plasma at 15 min (C*max* = 15.5 μg/mL). The study also revealed that the highest concentrations of betulin were found in the lungs and liver, with the lowest concentrations in the heart [186]. Another study using an LC–ESI/MS/MS system demonstrated that betulin (500 mg/kg) had a slight oral absorption and slow elimination rate in rats (C*max* = 59.6 ± 23.0 ng/mL, t1/2 = 16.9 ± 2.7 h, and MRT = 26.3 ± 7.6 h) [184]. A recent study of betulin metabolism in rats (100 mg/kg orally) identified 62 metabolites, including phase I and II metabolites, in samples of urine, bile, and plasma [183].

## 7. Limitations and Future Considerations

Although betulin exhibits a variety of pharmacological activities, from antiviral to antimicrobial, a recurrent drawback seen in both preclinical and clinical studies is the insolubility of betulin in aqueous solutions. These investigations all share the use of a vehicle including oils, DMSO (dimethyl sulfoxide), nanocarriers, oleogels, etc., corroborating the lipophilic nature of betulin. According to a pharmacokinetic study, betulin was only partially absorbed in the plasma of dogs and rats and had a solubility of less than 0.10 μg/mL [185]. It is possible to argue that betulin’s chemical makeup and structure, specifically its pentacyclic lupane carbon structure, are to blame for the substance’s low bioavailability and distribution. In an effort to increase the solubility and bioavailability of betulin, betulinic acid, a more soluble derivative, has been thoroughly explored. Also, in recent years, research has mainly focused on the chemical modification of betulin with ligands; synthesis of water-soluble derivatives; targeted delivery of betulin with nanocarriers such as nanoemulsion; and preparation of cholesterol-containing betulin liposomes. These methods may represent a paradigm shift in the successful management of viral infections and chronic diseases in clinical studies with betulin and its derivatives. This is because the preliminary results of different clinical trials of betulin-based oleogels against actinic keratosis suggest that betulin is an efficacious topical treatment for skin diseases [162,163]. Hence, more research is required on preclinical studies of more solubilized forms of betulin, the pharmacological effects and molecular mechanisms of these soluble derivatives, and the standardization and optimization of betulin concentrations.

Betulin derivatives have shown promising antiviral activity against SARS-CoV via targeting its main protease activities [187] which, along with other natural compounds with well-known anti-viral activity, such as coumarins [188]. Nevertheless, the well-established anti-inflammatory and antioxidant activities of betulin could be a valuable addition to the treatment of COVID-19-associated inflammation and secondary diseases as a combinatory approach [189]. 

There is also a need for more preclinical studies on the optimization of effective or inhibitive doses or concentrations, especially given the limited and insufficient resources available on the pharmacokinetics of betulin, despite studies claiming the non-toxicity of betulin *in vivo* even at a dose of 540 mg/kg [185]. As is the case with most therapeutics, the doses and concentrations employed in different studies significantly impact their outcomes. Although many studies have described betulin’s potential therapeutic effects *in vitro* and *in vivo*, little research has been performed on the oral/intravenous administration of betulin in human clinical trials for chronic diseases. Additionally, most clinical trials on betulin (Table 3) have focused on its ability to treat skin diseases and heal wounds since inflammation is a key process in wound healing. In addition, it seems that whether betulin exhibits inflammatory or anti-inflammatory properties is dependent on the concentration, incubation time, cell or tissue type (primary or cancer cell), and experimental setup (stimulated or unstimulated system). Therefore, in addition to its wound-healing capacity, there is a need for preliminary clinical trials, randomized large cohort groups, and controlled long-term trials for specific clinical applications of betulin since not all results from preclinical studies are applicable to humans. Moreover, because the inflammatory response is thought to play a role in the pathology of several chronic diseases, most *in vivo* experimental models employ different experimental designs, which can yield inconsistent results. This is especially true when data from various experimental models are combined over a range of study lengths. The disparities in study models, duration, cell lines, age, species, and animal gender make it appear impossible to establish the results from all of these studies and correlate them.

## 8. Conclusions

There is a growing research interest in natural products due to their numerous sources and pharmacological potential. Numerous clinical trials using betulin oleogel (proprietary name Episalvan) in the treatment of skin burns and epidermolysis bullosa have shown that its wound-healing ability is one of its notable features. This article highlights betulin’s cardioprotective, hepatoprotective, antidiabetic, anticancer, and protective effects against inflammation. The summarized research revealed that the multitarget anti-inflammatory properties of betulin could be the reason for its many beneficial effects in different diseases. Based on the curated data, preclinical studies have confirmed that betulin mediates different inflammatory-driven diseases by inhibiting ROS production, lessening pro-inflammatory cytokines, and improving anti-inflammatory cytokines. The underlying molecular mechanisms include increasing the expression of HO-1 and NQO1 by activating Nrf2, inhibiting NFκB, and inactivating MAPKs. Cumulative clinical studies have shown that betulin is beneficial for skin burns, actinic keratosis, and epidermolysis bullosa. For skin wounds, betulin promotes the initiation of the inflammatory phase of wound healing by transiently upregulating proinflammatory mediators and enhancing the migration of keratinocytes. All this knowledge contributes to our understanding of the role of betulin in disease, which may provide new potential therapeutic targets, but further research and preclinical studies are needed to explore its full range of pharmacological properties. Finally, pharmacokinetic, and toxicological studies of betulin also need to be explored, and improved methods to increase the solubility of betulin should be considered. 

## Figures and Tables

**Table 1:** Natural sources of betulin, their extraction solvents, and isolation techniques.

Sources	Parts	Solvent	Isolation Techniques	References
*Anemone raddeana*	Root	Ethanol	Solvent extraction	[10]
*Pyrola decorate* H.	Leaves	Acetidin/ethanol	Solvent extraction (maceration)	[11]
*Ougenia Dalbergioides*	Bark	Methanol	Solvent extraction	[12]
*Paecilomyces* WE3-F	Mycelial culture	Dichloromethane	Solvent extraction	[13]
*Betula pendula* Roth	Bark	Ethanol	Solvent extraction	[14]
*Pterodon emarginatus*	Stem bark	Ethanol	Solvent extraction	[15]
*Betula pendula* *Betula pubescens*	Bark, leaves	Ethanol	Pressurized liquid extraction	[16]
*Tectona grandis*	Stem bark	Methanol	Solvent extraction	[17]
*B. pendula* Roth	Outer bark	Chloroform/dichloromethane/ methanol	Solvent extraction (maceration)	[18]
*Schleichera oleosa* (Lour)	Bark	Ethanol	Solvent extraction	[19]
*Inonotus obliquus*	Mycelial culture	Isopropanol	Ultrasonic extraction	[20]
*Alnus glutinosa* (L.) Gaertn	Alder bark	CO_2_/ethanol	Supercritical fluid extraction	[21]
*Viscum album coloratum*	-	Ethanol	Solvent extraction	[22]
*Betula pendula*, Roth	Leaves	Ethanol	Solvent extraction (maceration)	[23]
*Asteracantha longifolia*	Leaves Stem	Ethanol	Solvent extraction	[24]
*Ligaria cuneifolia*	Aerial parts	Ethanol	Solvent extraction (maceration)	[25]
*Hedyotis hedyotidea*	Stem	-	-	[26]
*Quercus incana*	Leaves	Methanol	Solvent extraction	[27]
*Acacia nilotica*	Bark	Methanol	Solvent extraction (maceration)	[28]
*Rhizophora mucronata*	Leaves	Chloroform	Solvent extraction (Soxhlet)	[29]
*Euphorbia hyssopifolia* L.	Dried latex	Methanol	Solvent extraction (maceration)	[30]
*Baliospermum montanum*	Leaves	Methanol	Solvent extraction (Soxhlet)	[31]
*Fomes fomentarius*	Mycelium	Ethanol	Solvent extraction	[32]
*Matayba elaeagnoides*	Bark	Methanol	Solvent extraction (maceration)	[33]
*Croton bonplandianum*	-	Dichloromethane	Solvent extraction (maceration)	[34]
*Celtis sinensis*	Leaves	Hydrophobic DES	DES extraction	[35]
*Doliocarpus dentatus*	Leaves	Ethanolic extract	Solvent extraction (maceration)	[36]
*Acacia auriculiformis*	Stem bark	Ethyl acetate soluble fraction	Solvent extraction	[37]
*Xanthium sibiricum*	Roots	Methanol	-	[38]
*Pleurostylia capensis*	Bark/Root	Dichloromethane/methanol	Solvent extraction	[39]

**Table 3:** Hepatoprotective activity of betulin.

Experimental Model		Dose/Concentration	Pharmacological Indicators	Mechanism of Action	References
-	Wistar Rats (Ethanol-induced alcoholic steatohepatitis 4 g/kg for 8 weeks)	50 and 100 mg/kg b.wt.	TG ↓ ALP ↓ AST ↓ ALT ↓ TNF-α ↓ IL-1β ↓ TGF-β ↓ TBARs ↓ GSH ↑ ROS ↓	None	[88]
Hepatic stellate cells (LX-2 cells) ethanol 50 mM)	Male C57BL/6 mice (Ethanol 5 g/kg b.wt. 10 days)	6.25–25 μM 20 and 50 mg/kg	SREBP1 ↓ TG ↓ ALT ↓ AST ↓ p65 ↓ collagen-I ↓ α-SMA ↓	Sirt1/LKB1/AMPK signaling pathway	[89]
AML-12 cells (Ethanol 50 mM)	Male C57BL/6 mice (5 g/kg b.wt. EtOH for 4 weeks)	0–25 μM 20 and 50 mg/kg	SREBP 1 ↓ Lipin1 ↓ Lipin2 ↑ ALT ↓ AST ↓ IL-1α ↓ TG ↓ PPAR-α ↑ FASN ↓ PPAR-γ ↓ IL-1β↓ IL-6 ↓ TNF-α ↓ IL-18 ↓ caspase-1 ↓	blocking of P2X7/NLRP3 signaling pathway	[94]
Hepatic stellate cells (HSC-T6, EtOH 50 mM)	Male C57BL/6 mice (EtOH 5 mg/kg)	12.5–25 μM 20 or 50 mg/kg	ALT ↓ AST ↓ TG ↓ CYP2E1 ↓ SREBP-1c ↓ TLR4 ↓ p-STAT3 ↑ Collagen-I ↓ α-SMA ↓	TLR4 and STAT3 pathway	[91]
Rat liver stellate cell CFSC-2G (EtOH 50 mM)	-	10 μM	procollagen I ↓ TNF-α ↓ MMP2 ↓ TGF-β ↓ TIMP1/2 ↓ α-SMA ↓	inhibited NFκB and MAPKs signaling pathway	[95]
-	LPS/D-galactosamine-induced acute liver injury BALB/c mice	2–8 mg/kg b.wt.	MPO ↓ AST ↓ ALT ↓ TNF-α ↓ IL-1β ↓ PPARγ ↑	inhibiting the NFκB signaling pathway	[87]
Con A-stimulated splenocytes	Concanavalin A-challenged C57BL/6J mice	0–32 μg/mL 20 mg/kg	IFN-γ ↓ IL-4 ↓ IL-6 ↓ IL-10 ↓ IL-17 ↓ IL-2 ↓ ALT ↓ AST ↓ TNF-α ↓	None	[26]
-	Sprague Dawley rats (Cisplatin 10 mg/kg)	8 mg/kg	caspase 3 ↓ caspase 9 ↓ tBilirubin↓ albumin ↑ caspase 8 ↓ MDA ↓ TAC ↑ IL-1 ↓ AST ↓ ALT ↓ caspase 1 ↓ p53 ↓ Bax ↓ Bcl-2 ↑	NLRP3 pathway	[93]

N.B: ↑—upregulate/increase, ↓—downregulate/decrease.

**Table 4:** Molecular mechanisms of betulin on inflammatory mediators in different preclinical studies.

Experimental Model		Dose/Concentration	Pharmacological Indicator	Molecular Mechanism	References
-	λ-carrageenan-induced paw edema in male ICR mice	30 and 90 mg/kg	SOD ↑, GPx ↑ GR ↑ MDA ↓ NO ↓	None	[101]
-	Ovalbumin-induced asthma in female BALB/c mice	10 mg/kg	ROS ↓ SOD ↑ CAT ↑ GSH ↑ NO_2_ ↓ NO_3_ ↓ MDA ↓ IL-4 ↓ MMP-9 ↓ p65 ↓ TGF-β1 ↓ IL-5 ↓ IgE ↓ TNF-α ↓ IFN-γ ↑ TIMP-1 ↓ tTG ↓ IL-13 ↓ p-IκB-α ↓ TREM-1 ↓	Inhibited NFκB signaling axis	[102]
primary human keratinocytes	-	0.87 and 4.34 μg/mL	MIP-1α ↑ COX-2 ↑ IL-6 ↑ IL-8 ↑ IFN-γ ↑ IP-10 ↑ TNF-α ↑ RhoA ↑ MIP-1 β ↑ basic FGF ↑ RANTES ↑	Modulated RNA stability involving p38 MAPK	[103]
HepG2 U397 macrophages	-	0.5–10 µM	G6P ↓ PEPCK ↑ TNF-α ↓ IL-1β ↓	Glucocorticoid receptor-mediated pathway	[104]
thapsigargin induced endoplasmic reticulum stress in Hippocampal neuronal cells (HT-22)	-	10 µM	ROS ↓ HO-1 ↑ Bcl2 ↑ CHOP ↓ Caspase12 ↓ Cleaved caspase 3 ↓	mediated HO-1 induction	[32]
porcine chondrocytes	-	0.32 µg/L (4 weeks)	TGF-β1 ↑ BMP-7 ↓ IGF-1 ↑ type II collagen ↑ aggrecan ↑ decorin ↑ MMPs ↓ IL-1β ↓	None	[105]
-	Wild-type AB strain zebrafish	0.125–0.5 mg/mL	ROS ↓ IL-1β ↓ TNF-α ↓ Caspase 3 ↓ p38 α ↓ ERK1/2↓	ROS/MAPKs/NFĸB signaling axis	[106]
IL-1β Induced chondrocytes	C57BL/6 male wild-type (WT) mice	20 mg/kg/day i.p 0–200 μM	iNOS ↓ TNFα ↓ NO ↓ COX2 ↓ IL-6 ↓ PGE-2 ↓ Aggrecan ↑ Collagen II ↑ ADAMTS5 ↓ MMP13 ↓	inhibited NFκB activation	[107]

N.B: ↑—upregulate/increase, ↓—downregulate/decrease.

**Table 5:** Clinical studies of betulin.

Pathological Condition	Inclusion Criteria for Study Group	Duration	Compound	Mode of Administration	Intervention	Outcome of Study	References
Chronic hepatitis C: a pilot study	Patients between 20–71 years with serologically confirmed chronic hepatitis (ALT levels 1.5-fold than the upper normal limits before commencing the study) [n = 42]	12 weeks	Birch bark extract (betulin 75% and betulinic acid 3.5%)	Oral	8 doses of gelatine capsules/day (160 mg standardized extract per day) per capsule, 20 mg dry ethanol birch bark extract	Decreased level of ALT, HCV RNA, fatigue, and abdominal pain with the absence of dyspepsia	[161]
Treatment of actinic keratosis: a pilot study	Patients with between 1–10 flat actinic keratoses, barely hyperkeratotic, located in typical UV exposure hotspots [n = 28]	2 months	Birch bark ointment (betulin 80%)	Topical	Two treatment groups: Birch bark ointment only Combination therapy (Birch bark salve and cryotherapy)	clearing of lesions and anti-inflammatory	[162]
Randomized monocentric phase II study on actinic keratosis	patients older than 18 years having ≤ 10 actinic keratosis of both sexes [n = 15]	3 months	Betulin based oleogel	Topical	Two treatment groups: Betulin-based oleogel (2× daily) combination therapy with cryotherapy and betulin-based oleogel	Anti-inflammatory and antitumor activity	[163]
Phase III double-blind, randomized placebo-controlled trial on epidermolysis bullosa (EB) NCT03068780	Children with hereditary epidermolysis bullosa (≥4 years) and adults with EB target wounds (10 to 50 cm^2^ partial thickness wounds, aged between 21 days and 9 months) [n = 223]	90 days	Oleogel-S10 betulin 72–88% (Episalvan)	Topical	Treatment: Oleogel-S10 (90% sunflower oil, 10% birch bark extract) Placebo: sunflower oil gel	Enhanced wound healing	[164,165]
Split-thickness skin graft transplant: an open, blind-evaluated, controlled, prospective, randomized phase II trial	Inpatients (18–95 years) needing skin grafting because of trauma, chronic venous ulcers, burns, or surgical excision [n = 24]	14 days	Oleogel-S10	Topical	Two treatment groups: Oleogel-S10 + Mepilex dressing and Mepilex dressing only	Faster wound healing and reepithelization of split-thickness skin graft	[166]
Randomized, intra-individually controlled, open, blind evaluated, polycentric phase III study on superficial partial thickness burns (EudraCT No. 2012-000362-38)	Adults who have had two comparable burn wounds of greater than 40 cm^2^ and less than 12.5% of their total body surface area (TBSA) within 48 h after injury or a single superficial partial thickness burn wound of more than 80 cm^2^ and less than 25% of TBSA [n = 57].	3 months with 12 months post-injury follow-up	Oleogel-S10 (Episalvan)	Topical	Two intervention groups: Oleogel-S10 and Octenilin^®^ wound gel (Octenidine hydrochloride gel)	Accelerated wound healing and improved reepithelization	[167]

## Data Availability

Not applicable.

## Abbreviations

Activated protein 1 (AP1); alanine transaminase (ALT); adaptor protein (ASC); aspartate aminotransferase (AST); aspartate transaminase (AST); A disintegrin and metalloprotease with thrombospondin type I motifs (ADAMTS); basic fibroblast growth factor (basic FGF); blood urea nitrogen (BUN); Bone morphogenetic protein 7 (BMP7); catalase (CAT); c-Jun N-terminal kinase (JNK); creatine kinase (CK); electrocardiogram (ECG); extracellular signal regulated kinase 1 and 2 (ERK 1/2); glutathione (GSH); glutathione peroxidase (GPx); glutathione reductase (GR); heme oxygenase (HO); high mobility group box 1 (HMGB-1); inducible nitric oxide synthase (iNOS); interferon gamma (IFN-γ); IFN-γ-induced protein-10 (IP-10); insulin-like growth factor (IGF-1); interleukin 1 (IL1); interleukin 6 (IL6); lactate dehydrogenase (LDH); lipopolysaccharide (LPS); macrophage inflammatory protein-1α (MIP-1α); malondialdehyde (MDA); metalloproteinase-2 (MMP-2); MAPK protein phosphatase (MKP); mitogen-activated protein kinase (MAPK); myeloperoxidase (MPO); nitric oxide (NO); NLR family pyrin domain containing 3 (NLRP3); nuclear factor kappa-B (NFκB); peroxisome proliferator-activated receptor gamma (PPAR-γ); phosphoenolpyruvate carboxykinases (PEPCK); proliferating cell nuclear antigen (PCNA); protein kinase C (PKC); P2X purinoceptor 7 (P2X7r); reactive nitrogen species (RNS); reactive oxygen species (ROS); receptor activator of nuclear factor kappa-B ligand (RANKL); regulated upon activation, normal T-cell expressed, and secreted (RANTES); signal transducer and activator of transcription 3 (STAT3); smooth muscle α-actin (α-SMA); superoxide dismutase (SOD); the inflammatory cytokine tumor necrosis factor (TNF); threonine-x-tyrosine (TxY); tissue inhibitors of metalloproteinases (TIMP-1 and -2); toll-like receptor 4 (TLR4); transforming growth factor beta (TGF-β).

## References

[1] Amiri S., Dastghaib S., Ahmadi M., Mehrbod P., Khadem F., Behrouj H., Aghanoori M.R., Machaj F., Ghamsari M., Rosik J. (2020). Betulin and its derivatives as novel compounds with different pharmacological effects. Biotechnol. Adv..

[2] Hordyjewska A., Ostapiuk A., Horecka A., Kurzepa J. (2019). Betulin and betulinic acid: Triterpenoids derivatives with a powerful biological potential. Phytochem. Rev..

[3] Šiman P., Filipová A., Tichá A., Niang M., Bezrouk A., Havelek R. (2016). Effective method of purification of betulin from birch bark: The importance of its purity for scientific and medicinal use. PLoS ONE.

[4] Laavola M., Haavikko R., Hämäläinen M., Leppänen T., Nieminen R., Alakurtti S., Moreira V.M., Yli-Kauhaluoma J., Moilanen E. (2016). Betulin Derivatives Effectively Suppress Inflammation *in vitro* and *in vivo*. J. Nat. Prod..

[5] Zhao H., Liu Z., Liu W., Han X., Zhao M. (2016). Betulin attenuates lung and liver injuries in sepsis. Int. Immunopharmacol..

[6] Medzhitov R. (2008). Origin and physiological roles of inflammation. Nature.

[7] Chen L., Deng H., Cui H., Fang J., Zuo Z., Deng J., Li Y., Wang X., Zhao L. (2018). Inflammatory responses and inflammation-associated diseases in organs. Oncotarget.

[8] Hayek E.W.H., Jordis U., Moche W., Sauter F. (1989). A bicentennial of betulin. Phytochemistry.

[9] Tolstikov G.A., Flekhter O.B., Shultz E.E., Baltina L.A., Tolstikov A.G. (2005). Betulin and Its Derivatives. Chemistry and Biological Activity. Chem. Sustain. Dev..

[10] Yamashita K., Lu H., Lu J., Chen G., Yokoyama T., Sagara Y., Manabe M., Kodama H. (2002). Effect of three triterpenoids, lupeol, betulin, and betulinic acid on the stimulus-induced superoxide generation and tyrosyl phosphorylation of proteins in human neutrophils. Clin. Chim. Acta.

[11] Yang X., Peng Q., Liu Q., Hu J., Tang Z., Cui L., Lin Z., Xu B., Lu K., Yang F. (2017). Antioxidant activity against h2o2-induced cytotoxicity of the ethanol extract and compounds from pyrola decorate leaves. Pharm. Biol..

[12] Patel M.R., Rajput N., Panchal H.S., Dalwadi H.B. (2017). Quantification of Lupeol and Betulin in *Ougenia dalbergioides* Bark by Column Chromatography and TLC. J. Pharm. Sci. Biosci. Res..

[13] Barakat K., Saleh M. (2016). Bioactive Betulin produced by marine *Paecilomyces* WE3-F. J. Appl. Pharm. Sci..

[14] Drag M., Surowiak P., Malgorzata D.Z., Dietel M., Lage H., Oleksyszyn J. (2009). Comparision of the Cytotoxic Effects of Birch Bark Extract, Betulin and Betulinic Acid Towards Human Gastric Carcinoma and Pancreatic Carcinoma Drug-sensitive and Drug-Resistant Cell Lines. Molecules.

[15] De Moraes W.F., Galdino P.M., Nascimento M.V.M., Vanderlinde F.A., Bara M.T.F., Costa E.A., De Paula J.R. (2012). Triterpenes involved in the anti-inflammatory effect of ethanolic extract of *Pterodon emarginatus* Vogel stem bark. J. Nat. Med..

[16] Co M., Koskela P., Eklund-Åkergren P., Srinivas K., King J.W., Sjöberg P.J.R., Turner C. (2009). Pressurized liquid extraction of betulin and antioxidants from birch bark. Green Chem..

[17] Amol Singh P., Brindavanam N.B., Kimothi G.P., Verma R., Aeri V. (2016). A Validated HPLC method for the determination of betulin in the stem bark of *Tectona grandis* Linn. Int. J. Pharm. Sci. Res..

[18] Şoica C., Dehelean C., Peev C., Aluas M., Zupkó I., Kása P., Alexa E. (2012). Physico-chemical comparison of betulinic acid, betulin and birch bark extract and *in vitro* investigation of their cytotoxic effects towards skin epidermoid carcinoma (A431), breast carcinoma (MCF7) and cervix adenocarcinoma (HeLa) cell lines. Nat. Prod. Res..

[19] Khan M.J., Saraf S., Saraf S. (2017). Anti-inflammatory and associated analgesic activities of HPLC standardized alcoholic extract of known ayurvedic plant *Schleichera oleosa*. J. Ethnopharmacol..

[20] Bai Y.H., Feng Y.Q., Mao D.B., Xu C.P. (2012). Optimization for betulin production from mycelial culture of *Inonotus obliquus* by orthogonal design and evaluation of its antioxidant activity. J. Taiwan Inst. Chem. Eng..

[21] Felföldi-Gáva A., Szarka S., Simándi B., Blazics B., Simon B., Kéry Á. (2012). Supercritical fluid extraction of *Alnus glutinosa* (L.) Gaertn. J. Supercrit. Fluids.

[22] Ko B.S., Kang S., Moon B.R., Ryuk J.A., Park S. (2016). A 70% Ethanol Extract of Mistletoe Rich in Betulin, Betulinic Acid, and Oleanolic Acid Potentiated β-Cell Function and Mass and Enhanced Hepatic Insulin Sensitivity. Evid.-Based Complement. Altern. Med..

[23] Penkov D., Dimitrova S., Andonova V., Milieva E., Murdjeva M., Stanimirova I., Draganov M., Kassarova M. (2015). Biological activity of bulgarian folia betulae dry extract. Int. J. Pharm. Pharm. Sci..

[24] Misra T.N., Singh R.S., Pandey H.S., Singh B.K., Pandey R.P. (2001). Constituents of *Asteracantha longifolia*. Fitoterapia.

[25] Laiolo J., Barbieri C.L., Joray M.B., Lanza P.A., Palacios S.M., Vera D.M.A., Carpinella M.C. (2021). Plant extracts and betulin from *Ligaria cuneifolia* inhibit P-glycoprotein function in leukemia cells. Food Chem. Toxicol..

[26] Zhou Y., Weng X., Dou R., Tan X., Zhang T., Fang J., Wu X. (2017). Betulin from *Hedyotis hedyotidea* ameliorates concanavalin A-induced and T cell-mediated autoimmune hepatitis in mice. Acta Pharmacol. Sin..

[27] Zehra B., Ahmed A., Sarwar R., Khan A., Farooq U., Ali S.A., Al-Harrasi A. (2019). Apoptotic and antimetastatic activities of betulin isolated from *Quercus incana* against non-small cell lung cancer cells. Cancer Manag. Res..

[28] Kaur P., Arora S., Singh R. (2022). Isolation, characterization and biological activities of betulin from *Acacia nilotica* bark. Sci. Rep..

[29] Hridya V., Godson P., Chandrasekar N. (2012). Chromatographic identification of two biologically important triterpenoids from the chloroform extract of *Rhizophora mucronata*. Acta Chromatogr..

[30] Azaat A., Babojian G., Issa N. (2022). Phytochemical Screening, Antioxidant and Anticancer Activities of *Euphorbia hyssopifolia* L. against MDA-MB-231 Breast Cancer Cell Line. J. Turk. Chem. Soc. Sect. A Chem..

[31] Radhakrishna S., Kumari S.P. (2018). GCMS Analysis of total terpenoids from *Baliospermum montanum* and its antimicrobial activity. IAETSD J. Adv. Res. Appl. Sci..

[32] Lee S.O., Lee M.H., Lee K.R., Lee E.O., Lee H.J. (2019). *Fomes fomentarius* ethanol extract exerts inhibition of cell growth and motility induction of apoptosis via targeting AKT in human breast cancer MDA-MB-231 cells. Int. J. Mol. Sci..

[33] De Souza M.T., Buzzi F.D.C., Cechinel Filho V., Hess S., Delle Monache F., Niero R. (2007). Phytochemical and antinociceptive properties of *Matayba elaeagnoides* Radlk. barks. Z. Fur Naturforsch. Sect. C J. Biosci..

[34] Qaisar M.N., Uzair M., Imran M., Chaudhary B.A., Hussain S.N. (2016). New α-glucosidase inhibitors from *Croton bonplandianum Croton bonplandianum* Baill (Euphorbiaceae). Trop. J. Pharm. Res..

[35] Wang L., Hu Y., Guo G., Li J., Fang X., Zhao L. (2022). Enhanced and green extraction betulin from *Celtis sinensis* leaves using hydrophobic deep eutectic solvent. Biomass Convers. Biorefinery.

[36] Ishikawa R.B., Leitão M.M., Kassuya R.M., Macorini L.F., Moreira F.M.F., Cardoso C.A.L., Coelho R.G., Pott A., Gelfuso G.M., Croda J. (2017). Anti-inflammatory, antimycobacterial and genotoxic evaluation of *Doliocarpus dentatus*. J. Ethnopharmacol..

[37] Ahmadu A.A., Delehouzé C., Haruna A., Mustapha L., Lawal B.A., Udobre A., Baratte B., Triscornia C., Autret A., Robert T. (2021). Betulin, a newly characterized compound in *Acacia auriculiformis* bark, is a multi-target protein kinase inhibitor. Molecules.

[38] Ju A., Cho Y.C., Cho S. (2015). Methanol extracts of *Xanthium sibiricum* roots inhibit inflammatory responses via the inhibition of nuclear factor-κB (NF-κB) and signal transducer and activator of transcription 3 (STAT3) in murine macrophages. J. Ethnopharmacol..

[39] Razwinani M., Motaung K.S. (2022). The influence of friedelin, resinone, tingenone and betulin of compounds on chondrogenic differentiation of porcine adipose-derived mesenchymal stem cells (pADMSCs). Biochimie.

[40] Wexler R., Elton T., Pleister A., Feldman D. (2009). Cardiomyopathy: An Overview. Am. Fam. Physician.

[41] Ayyappan J.P., Lizardo K., Wang S., Yurkow E., Nagajyothi J.F. (2020). Inhibition of SREBP Improves Cardiac Lipidopathy, Improves Endoplasmic Reticulum Stress, and Modulates Chronic Chagas Cardiomyopathy. J. Am. Heart Assoc..

[42] Yu C., Cai X., Liu X., Liu J., Zhu N. (2021). Betulin Alleviates Myocardial Ischemia–Reperfusion Injury in Rats via Regulating the Siti1/NLRP3/NF-κB Signaling Pathway. Inflammation.

[43] Wen Y., Geng L., Zhou L., Pei X., Yang Z., Ding Z. (2020). Betulin alleviates on myocardial inflammation in diabetes mice via regulating Siti1/NLRP3/NF-κ-B pathway. Int. Immunopharmacol..

[44] Tang J.J., Li J.G., Qi W., Qiu W.W., Li P.S., Li B.L., Song B.L. (2011). Inhibition of SREBP by a small molecule, betulin, improves hyperlipidemia and insulin resistance and reduces atherosclerotic plaques. Cell Metab..

[45] Gui Y., Yan H., Gao F., Xi C., Li H., Wang Y. (2016). Betulin attenuates atherosclerosis in apoE^−/−^ mice by up-regulating ABCA1 and ABCG1. Acta Pharmacol. Sin..

[46] Muceniece R., Namniece J., Nakurte I., Jekabsons K., Riekstina U., Jansone B. (2016). Pharmacological research on natural substances in Latvia: Focus on lunasin, betulin, polyprenol and phlorizin. Pharmacol. Res..

[47] Ma C., Long H. (2016). Protective effect of betulin on cognitive decline in streptozotocin (STZ)-induced diabetic rats. Neurotoxicology.

[48] Alsaadi J.H.H. (2016). Isolation, Purification and Identification of Active Chemical Compound Lup-20(29)-ene-3, 28-diol (Betulin) from *Tetradium daniellii* Leaves and Study the hypoglycemic Effect on Rabbits. Univ. Thi-Qar J. Sci..

[49] Ratna Wulan D., Priyo Utomo E., Mahdi C. (2015). Antidiabetic Activity of *Ruellia tuberosa* L., Role of α -Amylase Inhibitor: *In Silico*, *In Vitro*, and *In Vivo* Approaches. Biochem. Res. Int..

[50] Wulan D.R., Utomo E.P., Mahdi C. (2014). Molecular modeling of *Ruellia tuberosa* L compounds as a-amylase inhibitor: An *in silico* comparation between human and rat enzyme model. Bioinformation.

[51] Thengyai S., Thiantongin P., Sontimuang C., Ovatlarnporn C., Puttarak P. (2020). α-Glucosidase and α-amylase inhibitory activities of medicinal plants in Thai antidiabetic recipes and bioactive compounds from *Vitex glabrata* R. Br. stem bark. J. Herb. Med..

[52] Gurupriya S., Cathrine L. (2021). Molecular docking studies of isolated compounds andrographolide and betulin from methanolic leaves extract of *Andrographis echioides* as alpha-amylase and alpha-glucosidase activators. Int. J. Appl. Pharm..

[53] Yuca H., Özbek H., Demirezer L., Güvenalp Z. (2022). Assessment of α-glucosidase and α-amylase inhibitory potential of *Paliurus spina-christi* Mill. and its terpenic compounds. Med. Chem. Res..

[54] Ilyina A., Arredondo-Valdés R., Farkhutdinov S., Segura-Ceniceros E.P., Martínez-Hernández J.L., Zaynullin R., Kunakova R. (2014). Effect of Betulin-containing Extract from Birch Tree Bark on α-Amylase Activity *In vitro* and on Weight Gain of Broiler Chickens *In vivo*. Plant Foods Hum. Nutr..

[55] Wardecki T., Werner P., Thomas M., Templin M.F., Schmidt G., Brandner J.M., Merfort I. (2016). Influence of Birch Bark Triterpenes on Keratinocytes and Fibroblasts from Diabetic and Nondiabetic Donors. J. Nat. Prod..

[56] Shafabakhsh R., Asemi Z. (2019). Quercetin: A natural compound for ovarian cancer treatment. J. Ovarian Res..

[57] Esposito S., Bianco A., Russo R., Di Maro A., Isernia C., Pedone P.V. (2019). Therapeutic Perspectives of Molecules from *Urtica dioica* Extracts for Cancer Treatment. Molecules.

[58] Imran M., Salehi B., Sharifi-Rad J., Gondal T.A., Saeed F., Imran A., Shahbaz M., Fokou P.V.T., Arshad M.U., Khan H. (2019). Kaempferol: A key emphasis to its anticancer potential. Molecules.

[59] Tuli H.S., Sak K., Gupta D.S., Kaur G., Aggarwal D., Parashar N.C., Choudhary R., Yerer M.B., Kaur J., Kumar M. (2021). Anti-inflammatory and anticancer properties of birch bark-derived betulin: Recent developments. Plants.

[60] Novío S., Cartea M.E., Soengas P., Freire-Garabal M., Núñez-Iglesias M.J. (2016). Effects of Brassicaceae isothiocyanates on prostate cancer. Molecules.

[61] Peng Y., Ao M., Dong B., Jiang Y., Yu L., Chen Z., Hu C., Xu R. (2021). Anti-Inflammatory Effects of Curcumin in the Inflammatory Diseases: Status, Limitations and Countermeasures. Drug Des. Dev. Ther..

[62] Barboza J.N., da Silva Maia Bezerra Filho C., Silva R.O., Medeiros J.V.R., de Sousa D.P. (2018). An overview on the anti-inflammatory potential and antioxidant profile of eugenol. Oxid. Med. Cell. Longev..

[63] Azab A., Nassar A., Azab A.N. (2016). Anti-inflammatory activity of natural products. Molecules.

[64] Man S., Gao W., Zhang Y., Huang L., Liu C. (2010). Chemical study and medical application of saponins as anti-cancer agents. Fitoterapia.

[65] Pyo J.S., Si H.R., Dae K.K., Jin G.L., Yong Y.L., Soon S.H., Sung W.K., Jeong H.P. (2009). Anti-cancer effect of betulin on a human lung cancer cell line: A pharmacoproteomic approach using 2 D SDS PAGE coupled with nano-HPLC tandem mass spectrometry. Planta Med..

[66] Gauthier C., Legault J., Lavoie S., Rondeau S., Tremblay S., Pichette A. (2009). Synthesis and cytotoxicity of bidesmosidic betulin and betulinic acid saponins. J. Nat. Prod..

[67] Dehelean C.A., Feflea S., Molnár J., Zupko I., Soica C. (2012). Betulin as an antitumor agent tested *in vitro* on A431, HeLa and MCF7, and as an angiogenic inhibitor *in vivo* in the CAM assay. Nat. Prod. Commun..

[68] Li Y., Liu X., Jiang D., Lin Y., Wang Y., Li Q., Liu L., Jin Y.H. (2016). Betulin induces reactive oxygen species-dependent apoptosis in human gastric cancer SGC7901 cells. Arch. Pharm. Res..

[69] Li Y., He K., Huang Y., Zheng D., Gao C., Cui L., Jin Y.H. (2010). Betulin induces mitochondrial cytochrome c release associated apoptosis in human cancer cells. Mol. Carcinog..

[70] Mullauer F.B., Kessler J.H., Medema J.P. (2009). Betulin is a potent anti-tumor agent that is enhanced by cholesterol. PLoS ONE.

[71] Rzeski W., Stepulak A., Szymański M., Juszczak M., Grabarska A., Sifringer M., Kaczor J., Kandefer-Szerszeń M. (2009). Betulin Elicits Anti-Cancer Effects in Tumour Primary Cultures and Cell Lines in vitro. Basic Clin. Pharmacol. Toxicol..

[72] Han Y.H., Mun J.G., Jeon H.D., Kee J.Y. (2020). Betulin inhibits lung metastasis by inducing cell cycle arrest, autophagy, and apoptosis of metastatic colorectal cancer cells. Nutrients.

[73] Dehelean C.A., Feflea S., Gheorgheosu D., Ganta S., Cimpean A.M., Muntean D., Amiji M.M. (2013). Anti-angiogenic and anti-cancer evaluation of betulin nanoemulsion in chicken chorioallantoic membrane and skin carcinoma in Balb/c mice. J. Biomed. Nanotechnol..

[74] Feng J.H., Duan X.Z., Pan J.Y., Li W.M., Zhang X.D., Zhang Y.S. (2017). Involvement of protein kinase C-δ activation in betulin-induced apoptosis of neuroblastoma. Trop. J. Pharm. Res..

[75] Oh S.-H., Choi J.-E., Lim S.-C. (2006). Protection of betulin against cadmium-induced apoptosis in hepatoma cells. Toxicology.

[76] Yim N.H., Jung Y.P., Kim A., Kim T., Ma J.Y. (2015). Induction of apoptotic cell death by betulin in multidrug-resistant human renal carcinoma cells. Oncol. Rep..

[77] Yin F., Feng F., Wang L., Wang X., Li Z., Cao Y. (2019). SREBP-1 inhibitor Betulin enhances the antitumor effect of Sorafenib on hepatocellular carcinoma via restricting cellular glycolytic activity. Cell Death Dis..

[78] Kvasnica M., Sarek J., Klinotova E., Dzubak P., Hajduch M. (2005). Synthesis of phthalates of betulinic acid and betulin with cytotoxic activity. Bioorganic Med. Chem..

[79] Gao H., Wu L., Kuroyanagi M., Harada K., Kawahara N., Nakane T., Umehara K., Hirasawa A., Nakamura Y. (2003). Antitumor-promoting constituents from *Chaenomeles sinensis* Koehne and their activities in JB6 mouse epidermal cells. Chem. Pharm. Bull..

[80] Taiyi S.J. (2010). Caspase-9 activation-critical for betulin-induced apoptosis of human hepatoma cells. Chem. Res. Chin. Univ..

[81] Zhang J., Zhou B., Sun J., Chen H., Yang Z. (2021). Betulin ameliorates 7,12-dimethylbenz(a)anthracene-induced rat mammary cancer by modulating MAPK and AhR/Nrf-2 signaling pathway. J. Biochem. Mol. Toxicol..

[82] Zhou Z., Zhu C., Cai Z., Zhao F., He L., Lou X., Qi X. (2018). Betulin induces cytochrome c release and apoptosis in colon cancer cells via NOXA. Oncol. Lett..

[83] Cheng W., Ji S., Zhang H., Han Z., Liu Q., Wang J., Ping H. (2017). mTOR activation is critical for betulin treatment in renal cell carcinoma cells. Biochem. Biophys. Res. Commun..

[84] Lin Y.C., Chen H.Y., Hsieh C.P., Huang Y.F., Chang I.L. (2020). Betulin inhibits mTOR and induces autophagy to promote apoptosis in human osteosarcoma cell lines. Environ. Toxicol..

[85] Yu J., Li M., Zhan D., Shi C., Fang L., Ban C., Zheng W., Veeraraghavan V., Mohan S., Tang X. (2020). Inhibitory effects of triterpenoid betulin on inflammatory mediators inducible nitric oxide synthase, cyclooxygenase-2, tumor necrosis factor-alpha, interleukin-6, and proliferating cell nuclear antigen in 1,2-dimethylhydrazine-induced rat colon carcinogenesis. Pharmacogn. Mag..

[86] Yang Q., Fei Z., Huang C. (2021). Betulin terpenoid targets OVCAR-3 human ovarian carcinoma cells by inducing mitochondrial mediated apoptosis, G2/M phase cell cycle arrest, inhibition of cell migration and invasion and modulating mTOR/PI3K/AKT signalling pathway. Cell. Mol. Biol..

[87] Xu G.M., Zan T., Li H.Y., Han J.F., Liu Z.M., Huang J., Dong L.H., Zhang H.N. (2018). Betulin inhibits lipopolysaccharide/D-galactosamine-induced acute liver injury in mice through activating PPAR-γ. Biomed. Pharmacother..

[88] Buko V., Kuzmitskaya I., Kirko S., Belonovskaya E., Naruta E., Lukivskaya O., Shlyahtun A., Ilyich T., Zakreska A., Zavodnik I. (2019). Betulin attenuated liver damage by prevention of hepatic mitochondrial dysfunction in rats with alcoholic steatohepatitis. Physiol. Int..

[89] Bai T., Yang Y., Yao Y.-L., Sun P., Lian L.-H., Wu Y.-L., Nan J.-X. (2016). Betulin alleviated ethanol-induced alcoholic liver injury via SIRT1/AMPK signaling pathway. Pharmacol. Res..

[90] Szuster-Ciesielska A., Plewka K., Kandefer-Szerszeń M. (2011). Betulin, betulinic acid and butein are inhibitors of acetaldehyde-induced activation of liver stellate cells. Pharmacol. Rep..

[91] Wan Y., Jiang S., Lian L.H., Bai T., Cui P.H., Sun X.T., Jin X.J., Wu Y.L., Nan J.X. (2013). Betulinic acid and betulin ameliorate acute ethanol-induced fatty liver via TLR4 and STAT3 *in vivo* and *in vitro*. Int. Immunopharmacol..

[92] Szuster-Ciesielska A., Kandefer-Szerszeń M. (2005). Protective effects of betulin and betulinic acid against ethanol-induced cytotoxicity in HepG2 cells. Pharmacol. Rep..

[93] Eisa N.H., El-Sherbiny M., Abo El-Magd N.F. (2021). Betulin alleviates cisplatin-induced hepatic injury in rats: Targeting apoptosis and Nek7-independent NLRP3 inflammasome pathways. Int. Immunopharmacol..

[94] Dou J.Y., Jiang Y.C., Hu Z.H., Yao K.C., Yuan M.H., Bao X.X., Zhou M.J., Liu Y., Li Z.X., Lian L.H. (2022). Betulin Targets Lipin1/2-Meidated P2X7 Receptor as a Therapeutic Approach to Attenuate Lipid Accumulation and Metaflammation. Biomol. Ther..

[95] Szuster-Ciesielska A., Plewka K., Daniluk J., Kandefer-Szerszeń M. (2011). Betulin and betulinic acid attenuate ethanol-induced liver stellate cell activation by inhibiting reactive oxygen species (ROS), cytokine (TNF-α, TGF-β) production and by influencing intracellular signaling. Toxicology.

[96] Koeberle A., Werz O. (2014). Multi-target approach for natural products in inflammation. Drug Discov. Today.

[97] Guo M.Y., Li W.Y., Zhang Z., Qiu C., Li C., Deng G. (2015). Betulin suppresses S. aureus-induced mammary gland inflammatory injury by regulating PPAR-γ in mice. Int. Immunopharmacol..

[98] Zhao H., Zheng Q., Hu X., Shen H., Li F. (2016). Betulin attenuates kidney injury in septic rats through inhibiting TLR4/NF-κB signaling pathway. Life Sci..

[99] Pfarr K., Danciu C., Arlt O., Neske C., Dehelean C., Pfeilschifter J.M., Radeke H.H. (2015). Simultaneous and dose dependent melanoma cytotoxic and immune stimulatory activity of betulin. PLoS ONE.

[100] Ra H.J., Lee H.J., Jo H.S., Nam D.C., Lee Y.B., Kang B.H., Moon D.K., Kim D.H., Lee C.J., Hwang S.C. (2017). Betulin suppressed interleukin-1β-induced gene expression, secretion and proteolytic activity of matrix metalloproteinase in cultured articular chondrocytes and production of matrix metalloproteinase in the knee joint of rat. Korean J. Physiol. Pharmacol..

[101] Lin W.Y., Sadhasivam S., Lin F.H. (2009). The dose dependent effects of betulin on porcine chondrocytes. Process Biochem..

[102] Kamaraj Y., Dhayalan S., Chinnaiyan U., Kumaresan V., Subramaniyan S., Kumar D., Muniyandi K., Punamalai G. (2021). Triterpenoid compound betulin attenuates allergic airway inflammation by modulating antioxidants, inflammatory cytokines and tissue transglutaminase in ovalbumin-induced asthma mice model. J. Pharm. Pharmacol..

[103] Ebeling S., Naumann K., Pollok S., Wardecki T., Vidal-y-Sy S., Nascimento J.M., Boerries M., Schmidt G., Brandner J.M., Merfort I. (2014). From a traditional medicinal plant to a rational drug: Understanding the clinically proven wound healing efficacy of birch bark extract. PLoS ONE.

[104] Ren L., Niu S., Sun Y., Liang Y., Zhao J., Zhang T., Zhang J. (2021). Anti-inflammatory action of betulin and its potential as a dissociated glucocorticoid receptor modulator. Food Chem. Toxicol..

[105] Lin W.-Y., Lin F.-H., Sadhasivam S., Savitha S. (2010). Antioxidant effects of betulin on porcine chondrocyte behavior in gelatin/C6S/C4S/HA modified tricopolymer scaffold. Mater. Sci. Eng. C.

[106] Ouyang T., Yin H., Yang J., Liu Y., Ma S. (2022). Tissue regeneration effect of betulin via inhibition of ROS/MAPKs/NF-ĸB axis using zebrafish model. Biomed. Pharmacother..

[107] Ren C., Jin J., Hu W., Chen Q., Yang J., Wu Y., Zhou Y., Sun L., Gao W., Zhang X. (2021). Betulin Alleviates the Inflammatory Response in Mouse Chondrocytes and Ameliorates Osteoarthritis via AKT/Nrf2/HO-1/NF-κB Axis. Front. Pharmacol..

[108] Bhattacharyya A., Chattopadhyay R., Mitra S., Crowe S.E. (2014). Oxidative stress: An essential factor in the pathogenesis of gastrointestinal mucosal diseases. Physiol. Rev..

[109] Ci X., Zhou J., Lv H., Yu Q., Peng L., Hua S. (2017). Betulin exhibits anti-inflammatory activity in lps-stimulated macrophages and endotoxin-shocked mice through an ampk/akt/nrf2-dependent mechanism. Cell Death Dis..

[110] Szuster-Ciesielska A., Pilipów K., Kandefer-Szerszeń M. (2010). Protective effect of betulin and betulinic acid on acetaminophen and ethanol-induced cytotoxicity and reactive oxygen species production in HepG2 cells. J. Pre-Clin. Clin. Res..

[111] Kruszniewska-Rajs C., Strzałka-Mrozik B., Kimsa-Dudek M., Synowiec-Wojtarowicz A., Chrobak E., Bębenek E., Boryczka S., Głuszek S., Gola J.M. (2022). The Influence of Betulin and Its Derivatives EB5 and ECH147 on the Antioxidant Status of Human Renal Proximal Tubule Epithelial Cells. Int. J. Mol. Sci..

[112] He F., Ru X., Wen T. (2020). NRF2, a transcription factor for stress response and beyond. Int. J. Mol. Sci..

[113] Keum Y.S., Choi B.Y. (2014). Molecular and chemical regulation of the keap1-Nrf2 signaling pathway. Molecules.

[114] Tu W., Wang H., Li S., Liu Q., Sha H. (2019). The anti-inflammatory and anti-oxidant mechanisms of the keap1/Nrf2/ARE signaling pathway in chronic diseases. Aging Dis..

[115] Zhang D.D. (2006). Mechanistic studies of the Nrf2-Keap1 signaling pathway. Drug Metab. Rev..

[116] Lee P.J., Park H.J., Yoo H.M., Cho N. (2019). Betulin Protects HT-22 Hippocampal Cells against ER Stress through Induction of Heme Oxygenase-1 and Inhibition of ROS Production. Nat. Prod. Commun..

[117] Arivazhagan L., Subramanian S.P. (2015). Tangeretin, a citrus flavonoid attenuates oxidative stress and protects hepatocellular architecture in rats with 7, 12-dimethylbenz(a)anthracene induced experimental mammary carcinoma. J. Funct. Foods.

[118] Zhu Y., Yang Q., Liu H., Song Z., Chen W. (2020). Phytochemical compounds targeting on Nrf2 for chemoprevention in colorectal cancer. Eur. J. Pharmacol..

[119] Ahmed S.M.U., Luo L., Namani A., Wang X.J., Tang X. (2017). Nrf2 signaling pathway: Pivotal roles in inflammation. Biochim. Biophys. Acta- Mol. Basis Dis..

[120] Loboda A., Rojczyk-Golebiewska E., Bednarczyk-Cwynar B., Zaprutko L., Jozkowicz A., Dulak J. (2012). Targeting Nrf2-mediated gene transcription by triterpenoids and their derivatives. Biomol. Ther..

[121] Loboda A., Jazwa A., Grochot-Przeczek A., Rutkowski A.J., Cisowski J., Agarwal A., Jozkowicz A., Dulak J. (2008). Heme oxygenase-1 and the vascular bed: From molecular mechanisms to therapeutic opportunities. Antioxid. Redox Signal..

[122] Shi X.Z., Lindholm P.F., Sarna S.K. (2003). NF-κB activation by oxidative stress and inflammation suppresses contractility in colonic circular smooth muscle cells. Gastroenterology.

[123] Hayden M.S., Ghosh S. (2004). Signaling to NF-κB. Genes Dev..

[124] Gilmore T.D. (2006). Introduction to NF-κB: Players, pathways, perspectives. Oncogene.

[125] Mitchell S., Vargas J., Hoffmann A. (2016). Signaling via the NFκB system. Wiley Interdiscip. Rev. Syst. Biol. Med..

[126] Monaco C., Andreakos E., Kiriakidis S., Mauri C., Bicknell C., Foxwell B., Cheshire N., Paleolog E., Feldmann M. (2004). Canonical pathway of nuclear factor κB activation selectively regulates proinflammatory and prothrombotic responses in human atherosclerosis. Proc. Natl. Acad. Sci. USA.

[127] Liu T., Zhang L., Joo D., Sun S.C. (2017). NF-κB signaling in inflammation. Signal Transduct. Target. Ther..

[128] Sethi G., Sung B., Aggarwal B.B. (2008). Nuclear factor-κB activation: From bench to bedside. Exp. Biol. Med..

[129] Wu Q., Li H., Qiu J., Feng H. (2014). Betulin protects mice from bacterial pneumonia and acute lung injury. Microb. Pathog..

[130] Sharif O., Bolshakov V.N., Raines S., Newham P., Perkins N.D. (2007). Transcriptional profiling of the LPS induced NF-κB response in macrophages. BMC Immunol..

[131] Kawai T., Akira S. (2007). Signaling to NF-κB by Toll-like receptors. Trends Mol. Med..

[132] El-Sherbiny M., Eisa N.H., Abo El-Magd N.F., Elsherbiny N.M., Said E., Khodir A.E. (2021). Anti-inflammatory/anti-apoptotic impact of betulin attenuates experimentally induced ulcerative colitis: An insight into TLR4/NF-kB/caspase signalling modulation. Environ. Toxicol. Pharmacol..

[133] Zhang S.Y., Zhao Q.F., Fang N.N., Yu J.G. (2015). Betulin inhibits pro-inflammatory cytokines expression through activation STAT3 signaling pathway in human cardiac cells. Eur. Rev. Med. Pharmacol. Sci..

[134] Clark R.B. (2002). The role of PPARs in inflammation and immunity. J. Leukoc. Biol..

[135] Simonin M.A., Bordji K., Boyault S., Bianchi A., Gouze E., Bécuwe P., Dauça M., Netter P., Terlain B. (2002). PPAR-γ ligands modulate effects of LPS in stimulated rat synovial fibroblasts. Am. J. Physiol.- Cell Physiol..

[136] Chunhua M., Long H., Zhu W., Liu Z., Jie R., Zhang Y., Wang Y. (2017). Betulin inhibited cigarette smoke-induced COPD in mice. Biomed. Pharmacother..

[137] Bellezza I., Mierla A.L., Minelli A. (2010). Nrf2 and NF-κB and their concerted modulation in cancer pathogenesis and progression. Cancers.

[138] Bellezza I., Grottelli S., Gatticchi L., Mierla A.L., Minelli A. (2014). α-Tocopheryl succinate pre-treatment attenuates quinone toxicity in prostate cancer PC3 cells. Gene.

[139] Sandberg M., Patil J., D’Angelo B., Weber S.G., Mallard C. (2014). NRF2-regulation in brain health and disease: Implication of cerebral inflammation. Neuropharmacology.

[140] Liu Q., Liu J.P., Mei J.H., Li S.J., Shi L.Q., Lin Z.H., Xie B.Y., Sun W.G., Wang Z.Y., Yang X.L. (2020). Betulin isolated from *Pyrola incarnata* Fisch. inhibited lipopolysaccharide (LPS)-induced neuroinflammation with the guidance of computer-aided drug design. Bioorganic Med. Chem. Lett..

[141] Shaul Y.D., Seger R. (2007). The MEK/ERK cascade: From signaling specificity to diverse functions. Biochim. Biophys. Acta-Mol. Cell Res..

[142] Raman M., Chen W., Cobb M.H. (2007). Differential regulation and properties of MAPKs. Oncogene.

[143] Pimienta G., Pascual J. (2007). Canonical and alternative MAPK signaling. Cell Cycle.

[144] Soares-Silva M., Diniz F.F., Gomes G.N., Bahia D. (2016). The mitogen-activated protein kinase (MAPK) pathway: Role in immune evasion by trypanosomatids. Front. Microbiol..

[145] Cuevas B.D., Abell A.N., Johnson G.L. (2007). Role of mitogen-activated protein kinase kinase kinases in signal integration. Oncogene.

[146] Morrison D.K. (2012). MAP kinase pathways. Cold Spring Harb. Perspect. Biol..

[147] Plotnikov A., Zehorai E., Procaccia S., Seger R. (2011). The MAPK cascades: Signaling components, nuclear roles and mechanisms of nuclear translocation. Biochim. Biophys. Acta-Mol. Cell Res..

[148] Yang Z., Zhang Q., Yu L., Zhu J., Cao Y., Gao X. (2021). The signaling pathways and targets of traditional Chinese medicine and natural medicine in triple-negative breast cancer. J. Ethnopharmacol..

[149] Yue J., López J.M. (2020). Understanding MAPK signaling pathways in apoptosis. Int. J. Mol. Sci..

[150] Zong X., Song D., Wang T., Xia X., Hu W., Han F., Wang Y. (2015). LFP-20, a porcine lactoferrin peptide, ameliorates LPS-induced inflammation via the MyD88/NF-κB and MyD88/MAPK signaling pathways. Dev. Comp. Immunol..

[151] O’Sullivan A.W., Wang J.H., Redmond H.P. (2009). NF-κB and P38 MAPK Inhibition Improve Survival in Endotoxin Shock and in a Cecal Ligation and Puncture Model of Sepsis in Combination With Antibiotic Therapy. J. Surg. Res..

[152] Wang Y., Shan X., Chen G., Jiang L., Wang Z., Fang Q., Liu X., Wang J., Zhang Y., Wu W. (2015). MD-2 as the target of a novel small molecule, L6H21, in the attenuation of LPS-induced inflammatory response and sepsis. Br. J. Pharmacol..

[153] Mizerska-Kowalska M., Sławinska-Brych A., Kaławaj K., Zurek A., Pawinska B., Rzeski W., Zdzisinska B. (2019). Betulin promotes differentiation of human osteoblasts *in vitro* and exerts an osteoinductive effect on the HfOB 1.19 cell line through activation of JNK, ERK1/2, and mTOR kinases. Molecules.

[154] Kim K.J., Lee Y., Hwang H.G., Sung S.H., Lee M., Son Y.J. (2018). Betulin suppresses osteoclast formation via down-regulating NFATc1. J. Clin. Med..

[155] Su C.H., Lin C.Y., Tsai C.H., Lee H.P., Lo L.C., Huang W.C., Wu Y.C., Hsieh C.L., Tang C.H. (2021). Betulin suppresses TNF-α and IL-1β production in osteoarthritis synovial fibroblasts by inhibiting the MEK/ERK/NF-κB pathway. J. Funct. Foods.

[156] Johnson G.L., Lapadat R. (2002). Mitogen-activated protein kinase pathways mediated by ERK, JNK, and p38 protein kinases. Science.

[157] Cicenas J., Zalyte E., Rimkus A., Dapkus D., Noreika R., Urbonavicius S. (2018). JNK, p38, ERK, and SGK1 inhibitors in cancer. Cancers.

[158] Martin D.E., Salzwedel K., Allaway G.P. (2008). Bevirimat: A Novel Maturation Inhibitor for the Treatment of HIV-1 Infection. Antivir. Chem. Chemother..

[159] Pârvănescu R.D., Watz C.G., Moacă E.A., Vlaia L., Marcovici I., Macașoi I.G., Borcan F., Olariu I., Coneac G., Drăghici G.A. (2021). Oleogel formulations for the topical delivery of betulin and lupeol in skin injuries—Preparation, physicochemical characterization, and pharmaco-toxicological evaluation. Molecules.

[160] Schwieger-Briel A., Kiritsi D., Schempp C., Has C., Schumann H. (2017). Betulin-based oleogel to improve wound healing in dystrophic epidermolysis bullosa: A prospective controlled proof-of-concept study. Dermatol. Res. Pract..

[161] Shikov A.N., Djachuk G.I., Sergeev D.V., Pozharitskaya O.N. (2011). Birch bark extract as therapy for chronic hepatitis C—A pilot study. Phytomedicine.

[162] Huyke C., Laszczyk M., Scheffler A., Ernst R., Schempp C.M. (2006). Treatment of actinic keratoses with birch bark extract: A pilot study. JDDG-J. Ger. Soc. Dermatol..

[163] Huyke C., Reuter J., Rödig M., Kersten A., Laszczyk M., Scheffler A., Nashan D., Schempp C. (2009). Treatment of actinic keratoses with a novel betulin-based oleogel. A prospective, randomized, comparative pilot study. JDDG-J. Ger. Soc. Dermatol..

[164] Kern J.S., Schwieger-Briel A., Löwe S., Sumeray M., Davis C., Martinez A.E. (2019). Oleogel-S10 Phase 3 study “EASE” for epidermolysis bullosa: Study design and rationale. Trials.

[165] Martinez A., Murrell D.F., Bruckner A.L., Kern J., Maher L., Cunningham T. (2022). BG01: Safety and efficacy of oleogel-S10 (birch triterpenes) for epidermolysis bullosa: Results of a 12-month interim analysis of the open-label phase from the EASE study. Br. J. Dermatol..

[166] Metelmann H.-R., Brandner J.M., Schumann H., Bross F., Fimmers R., Böttger K., Scheffler A., Podmelle F. (2015). Accelerated reepithelialization by triterpenes: Proof of concept in the healing of surgical skin lesions. Ski. Pharmacol. Physiol..

[167] Frew Q., Rennekampff H.O., Dziewulski P., Moiemen N., Zahn T., Hartmann B. (2019). Betulin wound gel accelerated healing of superficial partial thickness burns: Results of a randomized, intra-individually controlled, phase III trial with 12-months follow-up. Burns.

[168] Mao D.B., Feng Y.Q., Bai Y.H., Xu C.P. (2012). Novel biotransformation of betulin to produce betulone by *Rhodotorula mucilaginosa*. J. Taiwan Inst. Chem. Eng..

[169] Singh R. (2017). Microbial Biotransformation: A Process for Chemical Alterations. J. Bacteriol. Mycol. Open Access.

[170] Chrobak E., Bȩbenek E., Kadela-Tomanek M., Latocha M., Jelsch C., Wenger E., Boryczka S. (2016). Betulin phosphonates; synthesis, structure, and cytotoxic activity. Molecules.

[171] Król S.K., Bębenek E., Sławińska-Brych A., Dmoszyńska-Graniczka M., Boryczka S., Stepulak A. (2020). Synthetic betulin derivatives inhibit growth of glioma cells in vitro. Anticancer Res..

[172] Boryczka S., Bebenek E., Wietrzyk J., Kempińska K., Jastrzebska M., Kusz J., Nowak M. (2013). Synthesis, structure and cytotoxic activity of new acetylenic derivatives of betulin. Molecules.

[173] Yamansarov E.Y., Skvortsov D.A., Lopukhov A.V., Kovalev S.V., Evteev S.A., Petrov R.A., Klyachko N.L., Zyk N.V., Beloglazkina E.K., Ivanenkov Y.A. (2019). New ASGPR-targeted ligands based on glycoconjugated natural triterpenoids. Russ. Chem. Bull..

[174] Shcherban N.D., Mäki-Arvela P., Aho A., Sergiienko S.A., Skoryk M.A., Kolobova E., Simakova I.L., Eränen K., Smeds A., Hemming J. (2019). Preparation of Betulone Via Betulin Oxidation Over Ru Nanoparticles Deposited on Graphitic Carbon Nitride. Catal. Lett..

[175] Kumar D., Dubey K.K. (2017). An efficient process for the transformation of betulin to betulinic acid by a strain of *Bacillus megaterium*. 3 Biotech.

[176] Grishko V.V., Tarasova E.V., Ivshina I.B. (2013). Biotransformation of betulin to betulone by growing and resting cells of the actinobacterium *Rhodococcus rhodochrous* IEGM 66. Process Biochem..

[177] Feng Y., Li M., Liu J., Xu T.Y., Fang R.S., Chen Q.H., He G.Q. (2013). A novel one-step microbial transformation of betulin to betulinic acid catalysed by *Cunninghamella blakesleeana*. Food Chem..

[178] Liu H., Lei X.L., Li N., Zong M.H. (2013). Highly regioselective synthesis of betulone from betulin by growing cultures of marine fungus *Dothideomycete* sp. HQ 316564. J. Mol. Catal. B Enzym..

[179] Li J., Jiang B., Chen C., Fan B., Huang H., Chen G. (2019). Biotransformation of betulin by *Mucor subtilissimus* to discover anti-inflammatory derivatives. Phytochemistry.

[180] Chen Q.H., Liu J., Zhang H.F., He G.Q., Fu M.L. (2009). The betulinic acid production from betulin through biotransformation by fungi. Enzym. Microb. Technol..

[181] Hu Z., Wang Z., Luo S., James M.O., Wang Y. (2019). Phase II metabolism of betulin by rat and human UDP-glucuronosyltransferases and sulfotransferases. Chem. Biol. Interact..

[182] Zhang W., Jiang H., Yang J., Jin M., Du Y., Sun Q., Cao L., Xu H. (2019). Safety assessment and antioxidant evaluation of betulin by LC-MS combined with free radical assays. Anal. Biochem..

[183] Zhang W., Jiang H., Jin M., Wang Q., Sun Q., Du Y., Cao L., Xu H. (2018). UHPLC-Q-TOF-MS/MS based screening and identification of the metabolites *in vivo* after oral administration of betulin. Fitoterapia.

[184] Hu Z., Guo N., Wang Z., Liu Y., Wang Y., Ding W., Zhang D., Wang Y., Yan X. (2013). Development and validation of an LC-ESI/MS/MS method with precolumn derivatization for the determination of betulin in rat plasma. J. Chromatogr. B Anal. Technol. Biomed. Life Sci..

[185] Jäger S., Laszczyk M., Scheffler A. (2008). A Preliminary Pharmacokinetic Study of Betulin, the Main Pentacyclic Triterpene from Extract of Outer Bark of Birch (*Betulae alba* cortex). Molecules.

[186] Pozharitskaya O.N., Karlina M.V., Shikov A.N., Kosman V.M., Makarov V.G., Casals E., Rosenholm J.M. (2017). Pharmacokinetics and Tissue Disposition of Nanosystem-Entrapped Betulin After Endotracheal Administration to Rats. Eur. J. Drug Metab. Pharmacokinet..

[187] Alhadrami H.A., Sayed A.M., Sharif A.M., Azhar E.I., Rateb M.E. (2021). Olive-derived triterpenes suppress SARS-CoV-2 main protease: A promising scaffold for future therapeutics. Molecules.

[188] Sharapov A.D., Fatykhov R.F., Khalymbadzha I.A., Zyryanov G.V., Chupakhin O.N., Tsurkan M.V. (2023). Plant Coumarins with Anti-HIV Activity: Isolation and Mechanisms of Action. Int. J. Mol. Sci..

[189] Pokharkar O., Lakshmanan H., Zyryanov G., Tsurkan M. (2022). *In Silico* Evaluation of Antifungal Compounds from Marine Sponges against COVID-19-Associated Mucormycosis. Mar. Drugs.

